# Electrically switchable van der Waals magnon valves

**DOI:** 10.1038/s41467-021-26523-1

**Published:** 2021-11-01

**Authors:** Guangyi Chen, Shaomian Qi, Jianqiao Liu, Di Chen, Jiongjie Wang, Shili Yan, Yu Zhang, Shimin Cao, Ming Lu, Shibing Tian, Kangyao Chen, Peng Yu, Zheng Liu, X. C. Xie, Jiang Xiao, Ryuichi Shindou, Jian-Hao Chen

**Affiliations:** 1 International Center for Quantum Materials, School of Physics, Peking University, Beijing, China; 2 Beijing Academy of Quantum Information Sciences, Beijing, China; 3 Department of Physics and State Key Laboratory of Surface Physics, Fudan University, Shanghai, China; 4 Institute of Physics, Chinese Academy of Sciences, Beijing, China; 5 State Key Laboratory of Optoelectronic Materials and Technologies, School of Materials Science and Engineering, Sun Yat-sen University, Guangzhou, China; 6 School of Materials Science and Engineering, Nanyang Technological University, Singapore, Singapore; 7 CAS Center for Excellence in Topological Quantum Computation, University of Chinese Academy of Sciences, Beijing, China; 8 Key Laboratory for the Physics and Chemistry of Nanodevices, Peking University, Beijing, China; 9 Interdisciplinary Institute of Light-Element Quantum Materials and Research Center for Light-Element Advanced Materials, Peking University, Beijing, China

**Keywords:** Two-dimensional materials, Magnetic devices, Spintronics, Electronic and spintronic devices

## Abstract

Van der Waals magnets have emerged as a fertile ground for the exploration of highly tunable spin physics and spin-related technology. Two-dimensional (2D) magnons in van der Waals magnets are collective excitation of spins under strong confinement. Although considerable progress has been made in understanding 2D magnons, a crucial magnon device called the van der Waals magnon valve, in which the magnon signal can be completely and repeatedly turned on and off electrically, has yet to be realized. Here we demonstrate such magnon valves based on van der Waals antiferromagnetic insulator MnPS_3_. By applying DC electric current through the gate electrode, we show that the second harmonic thermal magnon (SHM) signal can be tuned from positive to negative. The guaranteed zero crossing during this tuning demonstrates a complete blocking of SHM transmission, arising from the nonlinear gate dependence of the non-equilibrium magnon density in the 2D spin channel. Using the switchable magnon valves we demonstrate a magnon-based inverter. These results illustrate the potential of van der Waals anti-ferromagnets for studying highly tunable spin-wave physics and for application in magnon-base circuitry in future information technology.

## Abstract (web summary)

A major challenge in magnon based approaches to information processing lies in developing valves to allow or supress the magnon signal. Here, Chen et al demonstrate a van der Waals magnet based magnon valve which can be tuned electrically over an exceptionally wide range.

## Introduction

Van der Waals magnets are recently discovered magnetic materials with covalent bonding within the two-dimensional atomic layers and van der Waals interactions between the layers^1–3^. Owing to the short-range nature of magnetic exchange interaction, van der Waals magnets usually have weak interlayer exchange coupling strengths^4^, making the spin system highly two-dimensional and susceptible to external perturbations^5^. Therefore, 2D magnons, quanta of spin waves propagating in van der Waals magnets, are highly tunable collective modes that are of great interest in fundamental science^6–9^ and are potentially technologically useful^10–12^. To harness static spin configurations and dynamic spin excitations for potential technological applications, spin valves, and magnon valves are two respective crucial types of devices^13,14^. Recently, spin valves based on van der Waals crystals with a high on–off ratio^15,16^ and electrical switchability^17,18^ have been demonstrated. The electrically switchable van der Waals magnon valve without varying external magnetic field, however, has yet to be realized.

Owing to the wave nature of magnons, creating “1” in a magnonic circuit (e.g., a state with finite signal) is relatively trivial, while creating “0” (e.g., a state with zero signal) is not. Using two diffusive magnon streams with the same magnitude and opposite directions, it is possible to create “0” at the detector electrode^19^. However, such an operation is closer to signal mixing rather than gating. On the other hand, making use of the concept of “gating” from the charge-based field effect transistor, it has been shown that the magnon conductivity of thin films of three-dimensional (3D) ferrimagnetic insulator yttrium iron garnet could indeed be tuned by passing a current through a strong spin-orbit coupling metal gate electrode^14^. The state of the art of such 3D magnon valve can achieve up to ~13% signal modulation^14^, which still falls short in terms of tunability. In this article, we report the realization of van der Waals magnon valves with 100% tunability using thin flakes of van der Waals antiferromagnetic insulator MnPS_3_.

## Results

**Fig. 1:**
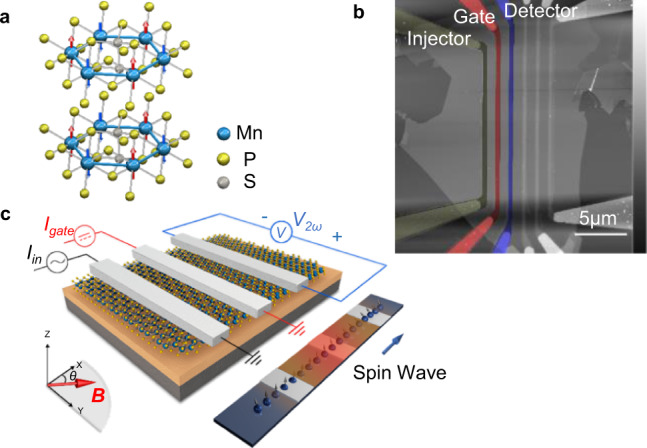
Basics of a MnPS_3_ magnon valve. **a** Atomic model of the crystal and spin structures of antiferromagnetic insulator MnPS_3_. **b** Atomic force micrograph of an MnPS_3_ magnon valve device. The injector, gate, and detector electrodes are marked by dark green, red, and blue. **c** Artistic schematics of the thermal magnon generation, manipulation, and detection. The upper left section shows the device structure with external circuits and direction of the external in-plane magnetic field; the lower right section shows propagation and modification of spin waves by the gate. Specifically, *I*_in_: AC injection current; *I*_gate_: DC gate current; *V*_*2ω*_: the second harmonic thermal magnon inverse spin Hall signal; *θ*: the angle of the in-plane magnetic field with respect to the *x* direction.

MnPS_3_ belongs to a class of layered antiferromagnetic insulators with chemical composition as XPS_y_ (*X* = Fe, Cr, Mn, Ni; *y* = 3, 4)^20–22^. It has an energy bandgap of ~3.0 eV for bulk crystals^23^ with an easy axis mostly perpendicular to the sample plane^22^. Within each layer, the manganese atoms form a hexagonal structure and the localized spin of ~5.9 μ_B_ on each manganese atom has antiferromagnetic exchange interaction with its nearest neighboring manganese atoms^22,24^, as shown in Fig. 1a. Between the layers, each manganese atom has its spin aligned in the same direction with the two manganese atoms directly below and above it. The ratio between in-plane and out-of-plane exchange coupling for the nearest neighbor Mn atoms is ~405:1^24^, making the magnons in MnPS_3_ highly two-dimensional.

Figure 1b shows the atomic force micrograph of a typical MnPS_3_ magnon valve device. The magnon valve constitutes the channel material MnPS_3_, an injector, a gate electrode, and a detector. The thickness of the particular MnPS_3_ channel is measured to be 12 nm and all electrodes are made of 250 nm wide and 9 nm thick platinum wires. A low-frequency AC current *I*_in_ is applied to the injector that locally heats up the MnPS_3_ crystal and thermally generates diffusive magnons. An in-plane magnetic field is applied that has a component perpendicular to the platinum detector electrode (defined as the *x* direction, see Fig. 1c) in order to tilt the out-of-plane spins towards such direction. In this configuration, the magnons would carry magnetic moments that can generate a second harmonic nonlocal voltage ${V}_{2\omega }$ at the platinum detector electrode via the inverse spin Hall effect^10,25^. An additional platinum electrode is fabricated between the injector and detector to act as a gate. We use a DC current *I*_*gate*_ applied through the gate electrode to control the signal at the detector. The schematics of the thermal magnon generation, manipulation, and detection are shown in Fig. 1c.

**Fig. 2:**
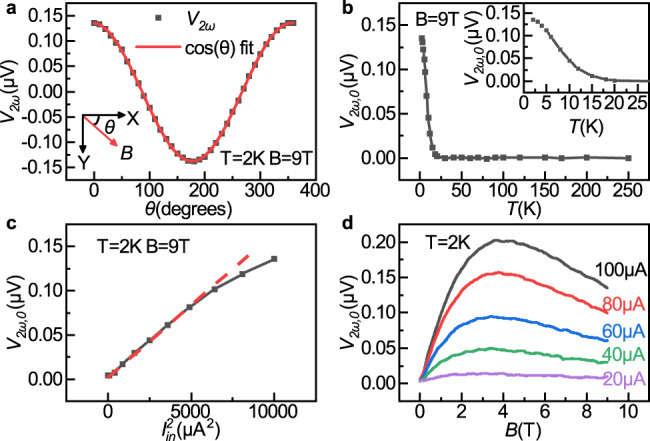
Second harmonic magnon signal *V*_2ω_ at zero gate current. **a** *V*_*2ω*_ as a function of the angle *θ* between the external magnetic field **B** and the *x* direction. Here angle *θ* is determined the same as shown in Fig. 1c. **b** Temperature dependence of *V*_*2ω*_ at *θ* = 0 (*V*_*2ω*,0_). (Inset: zoom-in view of *V*_*2ω*,0_ low-temperature behavior). **c** *V*_*2ω*,0_ versus the square of the injection current ${I}_{{{{{\rm{in}}}}}}^{2}$. **d** *V*_*2ω*,0_ versus **B** at different *I*_in_.

Figure 2a shows the magnetic field angle-dependent ${V}_{2\omega }$ of a MnPS_3_ magnon valve at *T* = 2 K. Here the gate electrode is electrically floating and no current is applied to it. The root mean square value of the injector current ${I}_{{in}}$ is 100 μA with a frequency of 18.07 Hz. The external magnetic field of 9 T is applied in-plane with an angle *θ* (*θ* = 0 when *H* is along the *x* direction, as shown in Fig. 1c). The red solid line in Fig. 2a is a fit to a cosine function. It can be seen that the ${V}_{2\omega }(\theta )$ data fits to the cosine function well with a 2π periodicity and a maximum value at $\theta =0$, consistent with previous studies on thermal magnons^10,26^. We simplify ${V}_{2\omega }(\theta =0)$ as ${V}_{2\omega ,0}$ in following description. Figure 2b shows the temperature dependence of the ${V}_{2\omega ,0}$. It can be seen that *V*_2ω,0_ does not appear until the sample is below 20 K, while the Néel temperature of MnPS_3_ is around 80 K^21^, consistent with previous study^26^. A recent study on thermal magnons in layered ferromagnet CrBr_3_ also shows similar behavior^27^. The inset in Fig. 2b shows a close up of the rising and saturation behavior of ${V}_{2\omega ,0}$ at low temperature.

Figure 2c shows the dependence of ${V}_{2\omega ,0}$ as a function of *I*_in_ at the injection electrode, which has a quadratic dependence ${V}_{2\omega ,0}\propto {I}_{{{{{\rm{in}}}}}}^{2}$ for *I*_in_ < 80 μA, as expected for signal from thermally generated magnons. The deviation from the quadratic relationship at *I*_in_ > 80 μA could be attributed to the increase of the sample temperature in the channel at the detector electrode. Figure 2d shows ${V}_{2\omega ,0}$ on **B** with different *I*_in_. ${V}_{2\omega ,0}$ initially increases linearly with **B**. This is consistent with the fact that the canting of the spins along the *x* direction is proportional to **B**, when **B** is small compares to the effective magnetic field of 106 T for the exchange interactions between the nearest neighboring Mn atoms^28^. At higher **B**, ${V}_{2\omega ,0}$ declines slightly, and the peak of ${V}_{2\omega ,0}$ appears to monotonically increase to higher **B** at higher *I*_in_, which could be owing to a suppression of the magnon diffusion length by external magnetic field^29^. It is worth noting that magnons injected by exchange interactions are absent (i.e., there is zero first harmonic nonlocal signal ${V}_{1\omega }(\theta )$ with a π periodicity to the angle of the in-plane magnetic field) in our MnPS_3_ devices (see Supplementary Fig. S6), which is consistent with previous studies^26^.

**Fig. 3:**
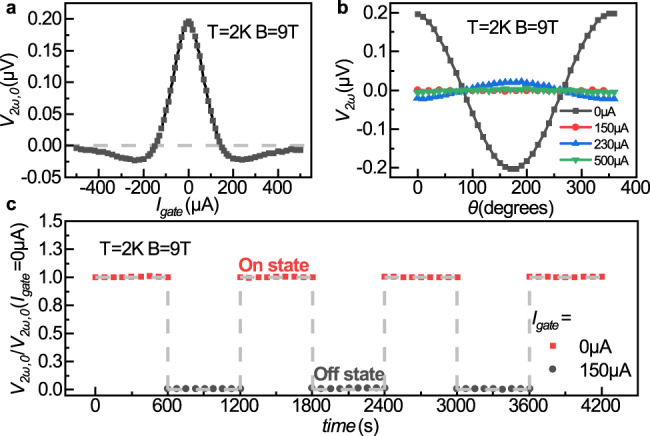
Operation of an MnPS_3_ magnon valve. **a** *V*_*2ω*,0_ versus DC gate current *I*_*gate*_ at *B* = 9 T and temperature of 2 K. **b** *V*_*2ω*_ as a function of angle *θ* of the external magnetic field at four different *I*_*gate*_ values. **c** Operation of the MnPS_3_ magnon valve with *I*_*gate*_ toggles repeatedly between 0 μA (On state) and 150 μA (Off state).

We now turn to the gating effect on the thermal magnon signal ${V}_{2\omega ,0}$ with **B** along the *x* direction. Figure 3a shows a typical dependence of the ${V}_{2\omega ,0}$ on the DC gate current *I*_*gate*_. First, it can be seen that ${V}_{2\omega ,0}$ is an even function of *I*_*gate*_, e.g., the effects of +*I*_*gate*_ and −*I*_*gate*_ are identical. Second, there exists a shut-off gate current *I*_*gate*_ = *I*_0_ that could completely suppress ${V}_{2\omega ,0}$. Remarkably, for *I*_*gate*_ = *I*_0_, ${V}_{2\omega ,0}$ become negative, and for a sufficiently large gate current ${I}_{{gate}}={I}_{0}^{{\prime} }$, ${V}_{2\omega ,0}$ tends to zero from the negative side. This means that the thermal magnon signal at the detector can be completely turned off by the gate, at two different values of *I*_*gate*_, which is ${I}_{0}$ and ${I}_{0}^{{\prime} }$. The existence of two zero points at the *V*_*2ω*,0_ (*I*_*gate*_) curve could be particularly useful for magnon logic operations. Among the two zero points, ${I}_{0}$ is more favorable since it is smaller and energetically more efficient.

Figure 3b shows the magnetic field angle-dependent ${V}_{2\omega }(\theta )$ for different *I*_*gate*_. The ${V}_{2\omega }(\theta )$ curves change sign between the two *I*_*gate*_ ranges: $0 < {I}_{{gate}} < {I}_{0}$ and ${I}_{0} < {I}_{{gate}} < {I}_{0}^{{\prime} }$. Furthermore, the ${V}_{2\omega }$ signal at ${I}_{{gate}}={I}_{0}$ and ${I}_{{gate}}={I}_{0}^{{\prime} }$ are suppressed completely for every angle *θ*. Such experimental observation proves that: (1) the sign reversal of ${V}_{2\omega ,0}$ observed in Fig. 3a can be completely attributed to the thermal magnon signal of the device; (2) the zero points of ${V}_{2\omega ,0}\left({I}_{{gate}}\right)$ observed in Fig. 3a are indeed zero points of the magnitude of the second harmonic magnon signal. Additional experiment and finite element analysis have also been carried out to rule out the possibility of a local spin Seebeck effect or an anomalous Nernst effect in our devices (details in Supplementary Information S9–S11). If one put ${V}_{2\omega }$ in a complex coordinate system (e.g., in the *x*+i*y* plane, where i is the imaginary unit), ${V}_{2\omega }$ initially located at the positive side of the *y* axis with zero gate; as the gate current increases, ${V}_{2\omega }$ continuously move to the origin along the *y* axis, reaching the origin at ${I}_{{gate}}={I}_{0}$, and continue to move towards the negative side of the *y* axis with a larger gate; with large enough gate current, e.g., ${I}_{{gate}}={I}_{0}{\prime}$, ${V}_{2\omega }$ asymptotically moves back to the origin of the complex 2D plane.

By repeatedly applying *I*_*gate*_ = 0 and *I*_*gate*_ = 150 μA, ${V}_{2\omega ,0}$ toggles between 196 nV (On state) and 0 nV (Off state), as shown in Fig. 3c. This demonstrates that the magnon valve can be electrically switched on and off without changing **B**. Such a fully electrical switching of the magnon signal lays the foundation of complex magnonic applications, such as logic gates that mimic those built from the charge-based transistors. In fact, Fig. 3c already illustrates the operation of a diffusive magnon-based NOT gate, which shows finite output (${V}_{2\omega ,0}=196{{{{{\rm{nV}}}}}}$) at zero input (*I*_*gate*_ = 0) and zero output (${V}_{2\omega ,0}=0$, here “0” means below the noise floor of our measurement system, which is < 1 nV) at finite input (*I*_*gate*_ = 150 μA).

In order to understand the gate-dependent behavior of ${V}_{2\omega ,0}$, it is necessary to look into the general form of the inverse spin Hall voltage *V*_ISHE_ at the detector electrode, which contains ${V}_{2\omega ,0}$ as it is excited by an AC current of frequency $\omega$. *V*_ISHE_ is proportional to the non-equilibrium magnon accumulation *n*_mag_ at the detector-MnPS_3_ interface^30,31^:

1 $${V}_{{ISHE}}\left({I}_{{in}}\left(t\right),{I}_{{gate}}\right)\propto {g}_{{mix}}{n}_{{mag}}\left(T\right).$$

Here, *g*_mix_ is the spin mixing conductance at the detector-MnPS_3_ interface which can be considered as a constant for our experimental conditions^32^. The non-equilibrium magnon accumulation *n*_mag_ is caused by the thermally driven magnon spin current ${{{{{{\bf{J}}}}}}}_{{{{{{\rm{m}}}}}}}$ along the *x* direction due to the Joule heating from the injector and the gate, which in turn is proportional to the lateral temperature gradient in the MnPS_3_ plane via the spin Seebeck effect^33–36^:

2 $${n}_{{mag}}\left(T\right)\propto \left|{{{{{{\bf{J}}}}}}}_{{{{{{\rm{m}}}}}}}(T)\right|\propto \left|{{{{{\bf{S}}}}}}(T)\cdot {{{{{\boldsymbol{\nabla }}}}}}{{{{{\rm{T}}}}}}\right|.$$

where the $2\times 2$ spin Seebeck coefficient tensor ${{{{{\bf{S}}}}}}$ is defined within the MnPS_3_ plane and is given below in Eq. (3). Since the temperature increase near the detector is proportional to the Joule heating from current applied in the injector and the gate, the magnon temperature ${T}=2K+\beta \left(\alpha {I}_{{{{{\rm{in}}}}}}^{2}+{I}_{{gate}}^{2}\right)$, where 2 K is the base temperature for the sample, and $\beta \left(\alpha {I}_{{{{{\rm{in}}}}}}^{2}+{I}_{{gate}}^{2}\right)$ accounts for the temperature increase due to the Joule heating from the injector and the gate. Here $\alpha\, < \, 1$ is a dimensionless geometrical factor to count for a larger distance of the injector than the gate to the detector, and $\beta$ is a constant involving the resistance of Pt bar and the specific heat of MnPS_3_. The lateral temperature gradient ${{{{{\boldsymbol{\nabla }}}}}}{{{{{\bf{T}}}}}}$ has a similar proportionality as ${{{{{\boldsymbol{\nabla }}}}}}{{{{{\bf{T}}}}}}\propto \beta (\alpha {I}_{{{{{\rm{in}}}}}}^{2}+{I}_{{gate}}^{2})\hat{{{{{{\bf{x}}}}}}}$.

The spin Seebeck coefficient tensor ${{{{{\bf{S}}}}}}$ in MnPS_3_ under in-plane magnetic field can be derived based on a semi-classical Boltzmann transport theory of 2D magnons (details at Supplementary information S3 and S4)^33^:

3 $${{{{{\bf{S}}}}}}(T)=\frac{{{{\hslash}}}^{2}{{\sin}}\psi}{{k}_{B}{T}^{2}}\mathop{\sum}\limits_{j\,=\,1,2}\int_{{BZ}}\frac{{dk}_{x}{dk}_{y}}{{\left(2\pi\right)}^{2}}{{{{{{\bf{v}}}}}}}_{{{{{{\rm{j}}}}}}}\left({{{{{\bf{k}}}}}}\right){{{{{{\bf{v}}}}}}}_{{{{{{\rm{j}}}}}}}\left({{{{{\bf{k}}}}}}\right){{\cosh}}{\xi}_{j}\frac{{e}^{{{\hslash}}{\omega}_{j}({{{{{\bf{k}}}}}})/{k}_{B}T}{\omega}_{j}({{{{{\bf{k}}}}}})}{{\eta}_{j,k}{\left({e}^{{{\hslash}}{\omega}_{j}({{{{{\bf{k}}}}}})/{k}_{B}T}-1\right)}^{2}}$$

where ${\eta }_{j,k}=1/{\tau }_{j,k}$, $\hslash {\omega }_{j}\left({{{{{\bf{k}}}}}}\right)$ and ${{{{{{\bf{v}}}}}}}_{{{{{{\rm{j}}}}}}}\left({{{{{\bf{k}}}}}}\right)$ are the magnon scattering rate, dispersion relation, and group velocity, respectively, for the *j*th magnon branch at magnon momentum **k**; $\psi$ is the canting angle of the spins from its easy axis at finite in-plane magnetic field, and ${{\sin }}\psi {{\cosh }}{\xi }_{j}$ is the *x*-component spin polarization of magnon density of the *j*th magnon branch (see Supplementary information S4).

Therefore, *V*_ISHE_ can be regarded as a function of $\beta \left(\alpha {I}_{{{{{\rm{in}}}}}}^{2}+{I}_{{gate}}^{2}\right)$, and the temporal dependence of *V*_ISHE_ comes purely from the time variation of ${I}_{{{{{\rm{in}}}}}}^{2}(t)$. Obviously, the temperature gradient ${{{{{\boldsymbol{\nabla }}}}}}{{{{{\bf{T}}}}}}$ increases monotonically with $\beta \left(\alpha {I}_{{{{{\rm{in}}}}}}^{2}+{I}_{{gate}}^{2}\right)$. The behavior of the spin Seebeck coefficient ${{{{{\bf{S}}}}}}\left(T\right)$ as shown in Eq. (3) is not so simple, but qualitatively ${{{{{\bf{S}}}}}}\left(T\right)$ should decrease as function of $\beta \left(\alpha {I}_{{{{{\rm{in}}}}}}^{2}+{I}_{{gate}}^{2}\right)$, because the elevated temperature would strongly reduce the magnon mean-free length. Consequently, the thermally driven magnon spin current ${{{{{{\bf{J}}}}}}}_{{{{{{\bf{m}}}}}}}$ will first increase, and then decrease with a general input current. In our real-time lock-in measurement, the first part will give a positive signal since more magnons are accumulating below the detector electrode with a non-zero input from the injection electrode. While in the second decreasing part, less magnons are accumulating below the detector electrode with applying the injection current, which equals to magnons flowing away from the detector electrode, resulting in a negative signal according to ISHE. The simulated functional dependence of *V*_ISHE_, ${{{{{\bf{S}}}}}}\left(T\right)$ and ${{{{{\boldsymbol{\nabla }}}}}}{{{{{\bf{T}}}}}}$ on a general input current can be found in Supplementary Fig. S4a; the temporal dependence and the frequency distribution of *V*_ISHE_ under an AC excitation are shown in Supplementary Fig. S4b and S4c, respectively.

With the above discussion, we arrive at the following equation from which the performance of the magnon valve devices at different *I*_in_ and *I*_*gate*_ can be simulated out of three global parameters:

4 $${V}_{2\omega ,0}=C * {\left[\beta \left(\alpha {I}_{{{{{\rm{in}}}}}}^{2}+{I}_{{gate}}^{2}\right) * S\left(T=2K+\beta \left(\alpha {I}_{{{{{\rm{in}}}}}}^{2}+{I}_{{gate}}^{2}\right)\right)\right]}_{2\omega }$$

where $C$, $\alpha$, $\beta$ are the three global parameters, and ${\left[{\ldots}\right]}_{2\omega }$ means taking the second harmonic component.

**Fig. 4:**
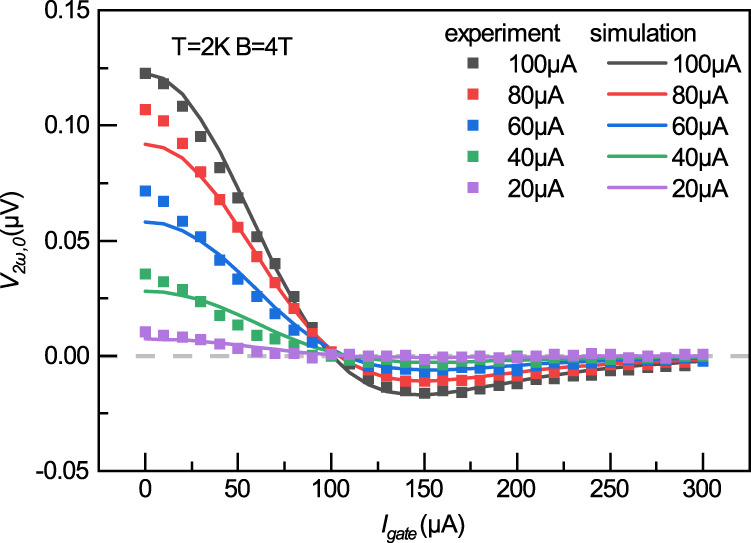
Gate tuning of a MnPS_3_ magnon valve with different *I*_in_: simulation and experiments. Simulation of gate-dependent *V*_*2ω,0*_ at 2 K and 4 T with five selected injection current *I*_in_. Only three global parameters are needed to produce all the simulation curves which match well with the experimental data.

Figure 4 shows the simulated ${V}_{2\omega ,0}{vs}.{I}_{{gate}}$ curves with *I*_*in*_ = 20, 40, 60, 80, 100 μA and ${{{{{\bf{B}}}}}}=4{{{{{\rm{T}}}}}}$ together with the experimental ${V}_{2\omega ,0}$ data under the same operation conditions. With just three global parameters in Eq. (4) (*C*, $\alpha$, and $\beta$), the simulation reproduces very well the experimental observation, including the naturally inferred symmetric ${V}_{2\omega ,0}$ for ±*I*_*gate*_, the magnitude of ${V}_{2\omega ,0}{vs}.{I}_{{gate}}$ under different *I*_in_, as well as the values of the zero point of ${V}_{2\omega ,0}$, e.g., *I*_0_ and ${I}_{0}^{{\prime} }$ vs different *I*_in_*.* The values of the global parameters $C=1.05\times {10}^{-26}{{{{{\rm{V}}}}}}\cdot {{{{{\rm{s}}}}}}/{{\hslash }}$, $\alpha =0.25$ and $\beta =1.69\times {10}^{-4}{{{{{\rm{K}}}}}}/{{{{{\rm{\mu }}}}}}{{{{{{\rm{A}}}}}}}^{2}$ are consistent with our experimental configuration, as detailed in Supplementary information S5. Furthermore, the simulation gives vanishingly small first harmonic response ${V}_{1\omega ,0}$, which agrees with the physics of thermal magnon excitation and it is indeed what we observed experimentally (see Supplementary Fig. S6). The above agreement between the simulation and the experimental data suggests that our model captures the physical trends behind the switching behavior of the MnPS_3_ magnon valves. The zero points at the thermal magnonic signal is guaranteed from our general argument, which comes from the highly tunable magnon spin current of the van der Waals antiferromagnetic insulators via electrical means. It is interesting to note that the first zero crossing point *I*_0_ is a weak function of the injection current *I*_in_, as can be seen in Fig. 4, which can be understood as the consequence of the steep initial slop and sharp peak of the inverse spin Hall voltage *V*_ISHE_ with an input current (as can be seen in Supplementary Fig. S4a). Indeed, the simulated zero crossing point *I*_0_ also reveals its weak but finite dependence on the injection current *I*_in_ (Supplementary Fig. S8). It is also worth noting that, albeit good overall agreement is found between the experimental data and theoretical simulation with only three global parameters, appreciable difference between the experiment and simulation is found for small *I*_*gate*_ (i.e., *I*_*gate*_ < 50 μA), which hints the existence of additional factors and warrants further study.

In conclusion, electrically controlled MnPS_3_-based magnon valves have been realized, in which the second harmonic magnon signal can be turned off by DC current through a metal gate. Such a zero crossing demonstrates a complete blocking of the transmission of the second harmonic magnon signal, enabled by the nonlinear gate dependence of the non-equilibrium magnon density of the van der Waals antiferromagnetic crystal. We expect that other van der Waals antiferromagnetic insulators with weak interlayer interactions would have very similar properties. Such strong and reversible electrical control of the magnon signal demonstrates the potential of van der Waals insulators with magnetic orders and paves the way to application of magnonics in future digital circuits.

## Methods

### Device fabrication and sample characterization

MnPS_3_ flakes are mechanically exfoliated from bulk crystals and deposited on 300 nm SiO_2_/Si substrates. Thickness of the MnPS_3_ flakes used in our study ranges from 10 nm to 30 nm. The injector, gate and detector electrodes in the magnon valves are fabricated with standard electron-beam lithography, platinum deposition and lift-off processes. Platinum is deposited in a magnetron sputtering system, and the width of the wires is ~250 nm with a thickness of 9 nm. Afterwards, 5 nm of titanium and 80 nm of gold are patterned to contact the platinum wires. Twelve devices (device 1–12) were made and studied. Data shown in the main text were obtained from device 1 (Fig. 1–3) and device 2 (Fig. 4), and the results for other devices are presented in the Supplementary Information.

### Nonlocal magnon transport measurement

The magnon transport measurement in MnPS_3_ is done in a physical properties measurement system (PPMS) with low-frequency lock-in amplifier technique. The injection AC current (18.07 Hz) in the range from 0 μA to 100 μA is provided by lock-in amplifier (NF LI5640) or a signal generator (Tektronix AFG 3000) with a 10KΩ resistor. Lock-in amplifiers (Stanford Research SR830) are used to probe the nonlocal voltages. Low noise voltage preamplifiers (NF LI75A) are also used. A voltage source (Keithley 2400) with a 100KΩ resistor is used to provide the DC current (0 μA~500 μA) to modulate the nonlocal signal. The temperature of the measurement in PPMS ranges from 2 K to 300 K, the applied magnetic field is parallel to our sample plane and the maximum field is 9 T.

## Supplementary information

Supplementary Information

Peer Review File

## Data Availability

Data for figures that support the current study are available at 10.7910/DVN/GLESFN. Source data are provided with this paper.

## Source data

Source data

## Code availability

Numerical simulations were performed with Mathematica. Source codes in Mathematica file format are available from the corresponding authors upon reasonable request. Source data are provided with this paper.
