# Supplementary material for: Electrically switchable van der Waals magnon valves
Source: Nat Commun. 2021 Nov 1;12:6279. doi: 10.1038/s41467-021-26523-1 (PMC8560771; doi:10.1038/s41467-021-26523-1)
Supplement: Supplementary file 1 — Supplementary Information [file 41467_2021_26523_MOESM1_ESM.pdf]

Supplementary information for
Electrically Switchable van der Waals Magnon Valves

Guangyi Chen[1†], Shaomian Qi[1†], Jianqiao Liu[1], Di Chen[1,2], Jiongjie Wang[3], Shili Yan[2], Yu Zhang[2], Shimin Cao[1,2], Ming Lu[1,2], Shibing Tian[4], Kangyao Chen[1], Peng Yu[5], Zheng Liu[6], X. C. Xie[1,2,7], Jiang Xiao[3], Ryuichi Shindou[1], Jian-Hao Chen[1,2,8,9*]

[1]*International Center of Quantum Materials, School of Physics, Peking University, Beijing, China*
[2]*Beijing Academy of Quantum Information Sciences, Beijing, China*
[3]*Department of Physics and State Key Laboratory of Surface Physics, Fudan University, Shanghai, China*
[4]*Institute of Physics, Chinese Academy of Sciences, Beijing, China*
[5] *State Key Laboratory of Optoelectronic Materials and Technologies, School of Materials Science and Engineering, Sun Yat-sen University, Guangzhou, China*
[6]*School of Materials Science and Engineering, Nanyang Technological University, Singapore, Singapore*
[7]*CAS Center for Excellence in Topological Quantum Computation, University of Chinese Academy of Sciences, Beijing 100190, China*
[8]*Key Laboratory for the Physics and Chemistry of Nanodevices, Peking University, Beijing, China*
[9]*Interdisciplinary Institute of Light-Element Quantum Materials and Research Center for Light-Element Advanced Materials, Peking University, Beijing*

[†]*These authors contributed equally to this work.*
[*]E-mail: *Jian-Hao Chen (chenjianhao@pku.edu.cn)*

Table of Contents:

S1. $V_{2\omega,0}(I_{gate})$ of a couple of $MnPS_3$ magnon valves at 2K

S2. $V_{2\omega,0}(I_{gate})$ of a typical $MnPS_3$ magnon valve at various temperatures

S3. Spin model and spin wave modes of $MnPS_3$ under an in-plane magnetic field

S4. Spin Seebeck effect in $MnPS_3$

S5. Quantitative analysis of the simulation

S6. Comparison between experimental and simulated $V_{1\omega}$ and $V_{2\omega}$

S7. Comparison between our work and recent work on $CrBr_3$

S8. The simulated crossing point I_0 vs. injection current I_{in}

S9. Nonlocal magnon signal detection with different $MnPS_3$ device geometries

S10. Absence of the anomalous Nernst effect in $MnPS_3$ magnon valve

S11. The heater-detector distance-dependent signal and temperature in $MnPS_3$ device

S12. Operation of a $MnPS_3$ magnon valve with different device geometries

S13. $R_{2\omega}$ vs. I_{in} for different $MnPS_3$ devices with zero gate current

S14. $V_{2\omega}$ vs. I_{in} for different $MnPS_3$ devices with zero gate current

S15. Stability test of few-layer $MnPS_3$ crystals and devices

S1. $V_{2\omega,0}(I_{gate})$ of a couple of $MnPS_3$ magnon valves at 2K

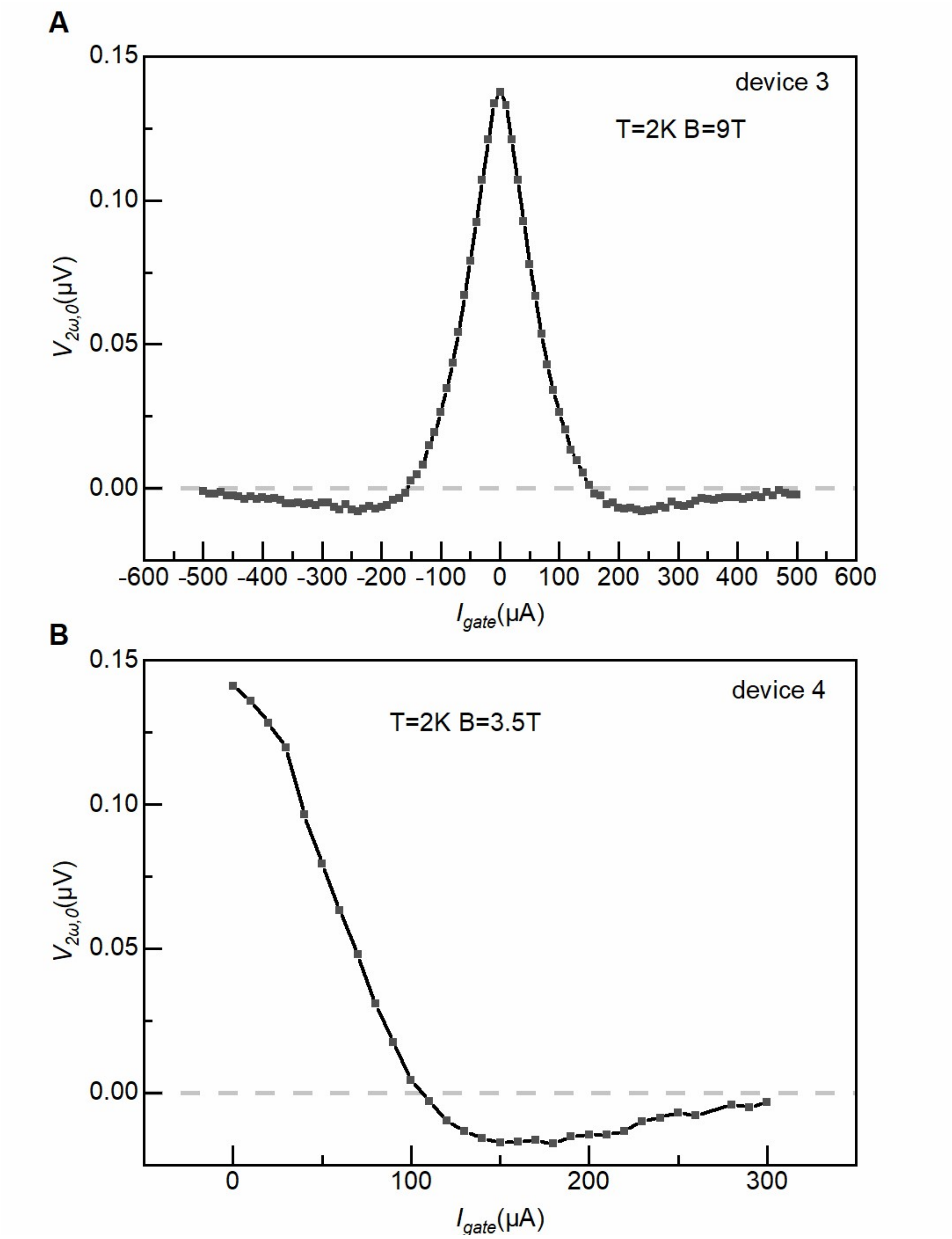

Fig. S1. $V_{2\omega,0}(I_{gate})$ of a couple of $MnPS_3$ magnon valves at 2K. $V_{2\omega,0}(I_{gate})$ of two more $MnPS_3$ magnon valves at 2K and at different in-plane magnetic fields.

S2. $V_{2\omega,0}(I_{gate})$ of a typical $MnPS_3$ magnon valve at various temperatures.

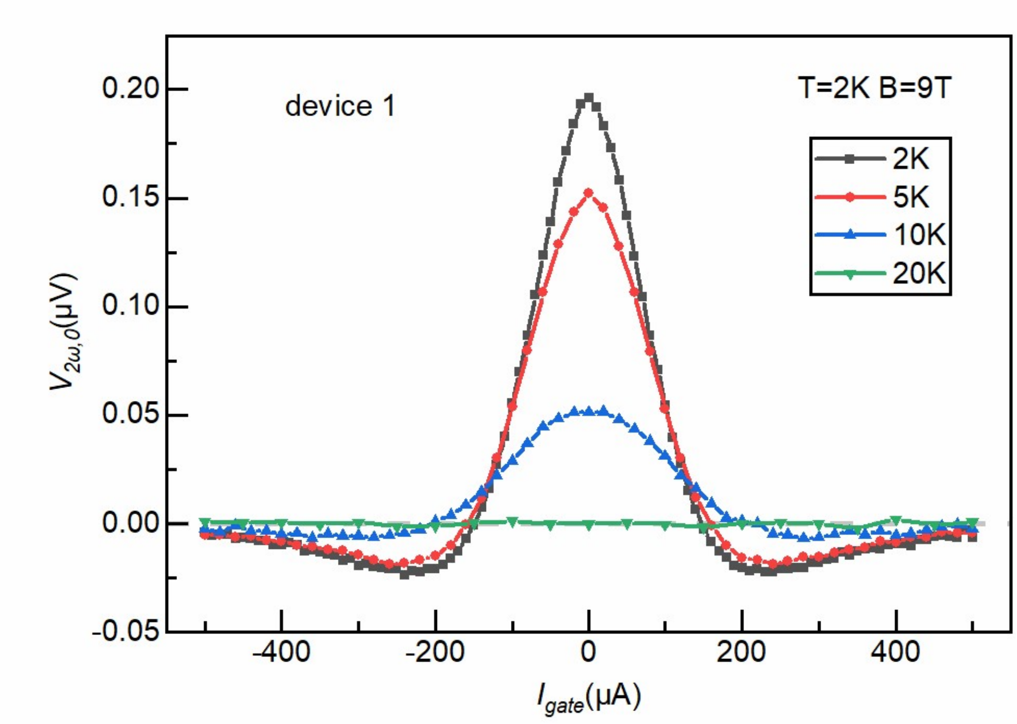

Fig. S2. $V_{2\omega,0}(I_{gate})$ of a typical $MnPS_3$ magnon valve at various temperatures. It can be seen that the higher the temperature, the weaker the $V_{2\omega,0}$ as well as its response to I_{gate}. For temperature higher than 20 K, $V_{2\omega,0}$ as well as its dependence on I_{gate} completely disappear, consistent with Fig. 2b in the main text.

S3. Spin model and spin wave modes of $MnPS_3$ under an in-plane magnetic field

In this section, we propose a two-dimensional localized spin model with easy-axis single-ion anisotropy to describe the antiferromagnetic insulator $MnPS_3$. We carry out spin-wave analysis of the Ising anti-ferromagnet under a transverse magnetic field to obtain low-energy magnon excitations.

Magnetism for the antiferromagnetic insulator $MnPS_3$ is described by a localized spin model with an easy-axis single-ion anisotropy.

$$H = J\sum_{\mathbf{j}}\sum_{m=1,2,3}\mathbf{S}_{\mathbf{j}}^{A}\cdot\mathbf{S}_{\mathbf{j+e_m}}^{B} - D\sum_{\mathbf{j}}\left[\left(S_{\mathbf{j}}^{A,z}\right)^2 + \left(S_{\mathbf{j+e_1}}^{B,z}\right)^2\right] - h\sum_{\mathbf{j}}\left[S_{\mathbf{j}}^{A,x} + S_{\mathbf{j+e_1}}^{B,x}\right] \quad \text{(S1)}$$

Here J is an antiferromagnetic exchange coupling and D is the easy-axis single-ion anisotropy. $\mathbf{j}$ denotes a honeycomb-lattice A-sublattice site, and $\mathbf{e}_m$ $(m = 1,2,3)$ connects a A-sublattice site and its neighboring three B-sublattice site in the honeycomb lattice, with $\mathbf{e}_1 = (1,0)$, $\mathbf{e}_2 = \left(-\frac{1}{2},\frac{\sqrt{3}}{2}\right)$, $\mathbf{e}_3 = \left(-\frac{1}{2},-\frac{\sqrt{3}}{2}\right)$. $\mathbf{S}_{j}^{\mathrm{A}} \equiv \left(S_{\mathbf{j}}^{\mathrm{A,x}}, S_{\mathbf{j}}^{\mathrm{A,y}}, S_{\mathbf{j}}^{\mathrm{A,z}}\right)$ is a localized spin of Mn atom (S=5/2)[1] in the A-sublattice site $(\mathbf{j})$ and $\mathbf{S}_{\mathbf{j}+\mathbf{e}_m}^{\mathrm{B}}$ is a localized Mn spin at the B-sublattice site$(\mathbf{j}+\mathbf{e}_m)$. For simplicity, we consider that the system is a two-dimensional magnet and h is an in-plane field along x-direction.

Under the in-plane field, the antiferromagnetic moment will be deformed linearly in the field:

$$\mathbf{S}_{\mathbf{j}}^{A} = S(\sin\psi, 0\cos\psi), \qquad \mathbf{S}_{\mathbf{j}}^{B} = S(\sin\psi, 0, -\cos\psi).$$

A canting angle is determined classically as a minimum of a classical magnetic energy:

$$E_{classical} = N(3JS^2(\sin^2\psi - \cos^2\psi) - 2DS^2\cos^2\psi - 2hS\sin\psi),$$

where N is a number of the A-sublattice sites. The minimum energy is given by:

$$\sin\psi = \frac{h}{(6J+2D)S} \quad \text{(S2)}$$

Magnetic collective excitations around the classical magnetic order are described by Holstein-Primakoff bosons;

$$\tilde{S}_{\mathbf{j}}^{A,z} = S - a_{\mathbf{j}}^{\dagger}a_{\mathbf{j}}, \qquad \tilde{S}_{\mathbf{j}}^{A,x} - i\tilde{S}_{\mathbf{j}}^{A,y} = \sqrt{2S}a_{\mathbf{j}}^{\dagger}, \qquad \tilde{S}_{\mathbf{j}}^{A,x} + i\tilde{S}_{\mathbf{j}}^{A,y} = \sqrt{2S}a_{\mathbf{j}}$$

$$\tilde{S}_{\mathbf{j}}^{B,z} = -S + b_{\mathbf{j}}^{\dagger}b_{\mathbf{j}}, \qquad \tilde{S}_{\mathbf{j}}^{B,x} + i\tilde{S}_{\mathbf{j}}^{B,y} = \sqrt{2S}b_{\mathbf{j}}^{\dagger}, \qquad \tilde{S}_{\mathbf{j}}^{B,x} - i\tilde{S}_{\mathbf{j}}^{B,y} = \sqrt{2S}b_{\mathbf{j}}$$

where $\left(\tilde{S}_{\mathbf{j}}^{A,x}, \tilde{S}_{\mathbf{j}}^{A,y}, \tilde{S}_{\mathbf{j}}^{A,z}\right)$ and $\left(\tilde{S}_{\mathbf{j}}^{B,x}, \tilde{S}_{\mathbf{j}}^{B,y}, \tilde{S}_{\mathbf{j}}^{B,z}\right)$ are the spin operators in a rotated frame;

$$\begin{pmatrix}\tilde{S}_{\mathbf{j}}^{A,x}\\ \tilde{S}_{\mathbf{j}}^{A,y}\\ \tilde{S}_{\mathbf{j}}^{A,z}\end{pmatrix} = \begin{pmatrix}\cos\psi & 0 & -\sin\psi\\ 0 & 1 & 0\\ \sin\psi & 0 & \cos\psi\end{pmatrix}\begin{pmatrix}S_{\mathbf{j}}^{A,x}\\ S_{\mathbf{j}}^{A,y}\\ S_{\mathbf{j}}^{A,z}\end{pmatrix}, \qquad \begin{pmatrix}\tilde{S}_{\mathbf{j}}^{B,x}\\ \tilde{S}_{\mathbf{j}}^{B,y}\\ \tilde{S}_{\mathbf{j}}^{B,z}\end{pmatrix} = \begin{pmatrix}\cos\psi & 0 & \sin\psi\\ 0 & 1 & 0\\ -\sin\psi & 0 & \cos\psi\end{pmatrix}\begin{pmatrix}S_{\mathbf{j}}^{B,x}\\ S_{\mathbf{j}}^{B,y}\\ S_{\mathbf{j}}^{B,z}\end{pmatrix}$$

a and b are Holstein-Primakoff boson fields for A-sublattice Mn spin and B-sublattice Mn spin, that represent fluctuations around the classical magnetic order. Around those ψ that minimize the classical magnetic energy, the Hamiltonian is stable against such small fluctuations:

$$H \equiv E_{classical} + H_{sw} + \mathcal{O}(a^3, b^3)$$

That says, a spin-wave Hamiltonian H_{sw} is given by a quadratic form in the boson fields:

$$H_{sw} = \frac{JS}{2}\cos 2\psi \sum_{j} \sum_{m=1,2,3} \left(a_{\mathbf{j}}^{\dagger} b_{\mathbf{j}+\mathbf{e}_m}^{\dagger} + a_{\mathbf{j}} b_{\mathbf{j}+\mathbf{e}_m} + a_{\mathbf{j}}^{\dagger} b_{\mathbf{j}+\mathbf{e}_m} + a_{\mathbf{j}} b_{\mathbf{j}+\mathbf{e}_m}^{\dagger}\right)$$
$$+ \frac{JS}{2} \sum_{j} \sum_{m=1,2,3} \left(a_{\mathbf{j}}^{\dagger} b_{\mathbf{j}+\mathbf{e}_m}^{\dagger} + a_{\mathbf{j}} b_{\mathbf{j}+\mathbf{e}_m} - a_{\mathbf{j}}^{\dagger} b_{\mathbf{j}+\mathbf{e}_m} - a_{\mathbf{j}} b_{\mathbf{j}+\mathbf{e}_m}^{\dagger}\right) - \frac{DS}{2} sin^2 \psi \sum_{\mathbf{j}} (a_{\mathbf{j}}^{\dagger} a_{\mathbf{j}}^{\dagger}$$
$$+ a_{\mathbf{j}} a_{\mathbf{j}} + a_{\mathbf{j}}^{\dagger} a_{\mathbf{j}} + a_{\mathbf{j}} a_{\mathbf{j}}^{\dagger} + b_{\mathbf{j}}^{\dagger} b_{\mathbf{j}}^{\dagger} + b_{\mathbf{j}} b_{\mathbf{j}} + b_{\mathbf{j}}^{\dagger} b_{\mathbf{j}} + b_{\mathbf{j}} b_{\mathbf{j}}^{\dagger}) + (3JS$$
$$+ 2DS) \sum_{\mathbf{j}} \left(a_{\mathbf{j}}^{\dagger} a_{\mathbf{j}} + b_{\mathbf{j}}^{\dagger} b_{\mathbf{j}}\right)$$

Here equation (S2) is used for replacing h by J and D. In the momentum space, the spin-wave Hamiltonian reads,

$$H_{sw} = \sum_{k} \left(a_{\mathbf{k}}^{\dagger} \quad b_{\mathbf{k}}^{\dagger} \quad a_{-\mathbf{k}} \quad b_{-\mathbf{k}}\right) \begin{pmatrix} M_0 & M_1 e^{i\varphi_{\mathbf{k}}} & -N_0 & N_1 e^{i\varphi_{\mathbf{k}}} \\ M_1 e^{-i\varphi_{\mathbf{k}}} & M_0 & N_1 e^{-i\varphi_{\mathbf{k}}} & -N_0 \\ -N_0 & N_{1e^{i\varphi_{\mathbf{k}}}} & M_0 & M_1 e^{i\varphi_{\mathbf{k}}} \\ N_1 e^{-i\varphi_{\mathbf{k}}} & -N_0 & M_1 e^{-i\varphi_{\mathbf{k}}} & M_0 \end{pmatrix} \begin{pmatrix} a_{\mathbf{k}} \\ b_{\mathbf{k}} \\ a_{-\mathbf{k}}^{\dagger} \\ b_{-\mathbf{k}}^{\dagger} \end{pmatrix}$$
$$= M_0 \sigma_0 \otimes \tau_0 + M_1 \sigma_0 \otimes (\cos \varphi_{\mathbf{k}} \tau_1 - \sin \varphi_{\mathbf{k}} \tau_2) - N_0 \sigma_1 \otimes \tau_0 + N_1 \sigma_1$$
$$\otimes (\cos \varphi_{\mathbf{k}} \tau_1 - \sin\varphi_{\mathbf{k}} \tau_2)$$

with $M_0 = \frac{3JS}{2} + DS - \frac{DS}{2}\sin^2\psi$, $M_1 = \frac{JS}{4}|f(k)|(-1+\cos 2\psi)$, $N_0 = \frac{DS}{2}\sin^2\psi$, $N_1 = \frac{JS}{4}|f(\mathbf{k})|(1+\cos 2\psi)$, $|f(\mathbf{k})|$ and $\varphi_{\mathbf{k}}$ are the modulus and phase of $f(\mathbf{k}) = \sum_{m=1,2,3} e^{ik\hat{e}_m} \equiv |f(\mathbf{k})| e^{i\varphi_{\mathbf{k}}}$ respectively, with $\varphi_{-\mathbf{k}} = -\varphi_{\mathbf{k}}$.

Under

$$\begin{pmatrix} a_{\mathbf{k}} \\ b_{\mathbf{k}} \\ a_{-\mathbf{k}}^{\dagger} \\ b_{-\mathbf{k}}^{\dagger} \end{pmatrix} = \sigma_0 \otimes \frac{1}{\sqrt{2}} \begin{pmatrix} e^{i\varphi_{\mathbf{k}}/2} & 0 \\ 0 & e^{-i\varphi_{\mathbf{k}}/2} \end{pmatrix} \begin{pmatrix} 1 & -1 \\ 1 & 1 \end{pmatrix} \begin{pmatrix} \alpha_{\mathbf{k}} \\ \beta_{\mathbf{k}} \\ \alpha_{-\mathbf{k}}^{\dagger} \\ \beta_{-\mathbf{k}}^{\dagger} \end{pmatrix},$$

$$\begin{pmatrix} \alpha_{\mathbf{k}} \\ \alpha_{-\mathbf{k}}^{\dagger} \end{pmatrix} = \begin{pmatrix} \cosh\frac{\xi_1}{2} & -\sinh\frac{\xi_1}{2} \\ -\sinh\frac{\xi_1}{2} & \cosh\frac{\xi_1}{2} \end{pmatrix} \begin{pmatrix} \gamma_{1,\mathbf{k}} \\ \gamma_{1,-\mathbf{k}}^{\dagger} \end{pmatrix}, \quad \begin{pmatrix} \beta_{\mathbf{k}} \\ \beta_{-\mathbf{k}}^{\dagger} \end{pmatrix} = \begin{pmatrix} \cosh\frac{\xi_2}{2} & \sinh\frac{\xi_2}{2} \\ \sinh\frac{\xi_2}{2} & \cosh\frac{\xi_2}{2} \end{pmatrix} \begin{pmatrix} \gamma_{2,\mathbf{k}} \\ \gamma_{2,-\mathbf{k}}^{\dagger} \end{pmatrix},$$

The spin-wave Hamiltonian is diagonalized as,

$$H_{sw} = \sum_k (\hbar\omega_1(\mathbf{k})\gamma^{\dagger}_{1,\mathbf{k}}\gamma_{1,\mathbf{k}} + \hbar\omega_2(\mathbf{k})\gamma^{\dagger}_{2,\mathbf{k}}\gamma_{2,\mathbf{k}}) .$$

Here $\cosh\xi_1 = \frac{M_0+M_1}{\hbar\omega_1(\mathbf{k})}$, $\sinh\xi_1 = \frac{-N_0+N_1}{\hbar\omega_1(\mathbf{k})}$, $\cosh\xi_2 = \frac{M_0-M_1}{\hbar\omega_2(\mathbf{k})}$, $\sinh\xi_2 = \frac{N_0+N_1}{\hbar\omega_2(\mathbf{k})}$, $\hbar\omega_1(\mathbf{k}) = \sqrt{(M_0+M_1)^2-(N_0-N_1)^2}$, $\hbar\omega_2(\mathbf{k}) = \sqrt{(M_0-M_1)^2-(N_0+N_1)^2}$, with $\omega_1(\mathbf{k}) < \omega_2(\mathbf{k})$.

The two spin-wave energy-momentum dispersions along high symmetric momentum line are plotted in Fig. S3(A).

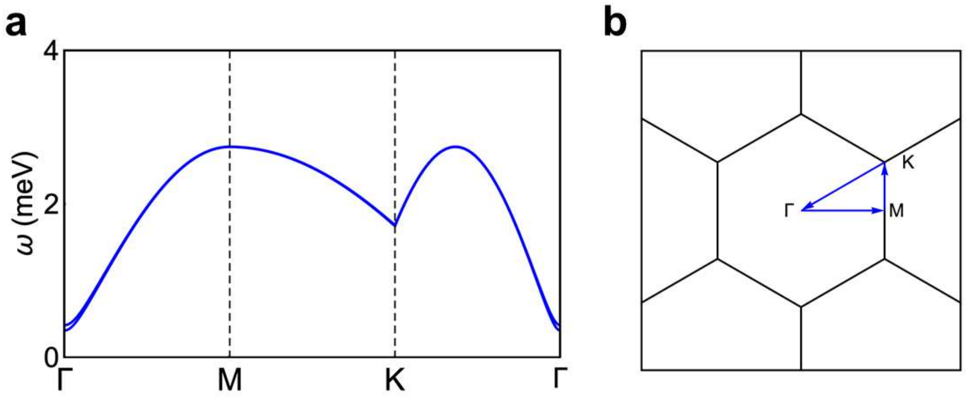

Fig. S3. Spin model and spin wave modes of $MnPS_3$ under an in-plane magnetic field. (a) The dispersions relation of spin waves along high symmetry directions of the $MnPS_3$ crystal when the in-plane nearest neighbor coupling J = 0.77meV, the magnetic anisotropy D = 0.0086meV and an in-plane magnetic field along the x direction of 4T. (b) Illustration of the high symmetry directions in the Brillouin zone of Mn honeycomb lattice in $MnPS_3$.

S4. Spin Seebeck effect in $MnPS_3$

Based on a semi-classical Boltzmann transport theory of magnon in anti-ferromagnet[2], we will give an expression for spin Seebeck coefficient of $MnPS_3$ at finite temperature and finite magnetic field in this section.

The spin density along the field direction is given by magnon creation and annihilation operators:

$$\sum_{\mathbf{j}}\left(S_{\mathbf{j}}^{A,x}+S_{\mathbf{j}}^{B,x}\right)=\sum_{\mathbf{j}}\left(\cos\psi\,\tilde{S}_{\mathbf{j}}^{A,x}+\sin\psi\,\tilde{S}_{\mathbf{j}}^{A,z}+\cos\psi\,\tilde{S}_{\mathbf{j}}^{B,x}-\sin\psi\,\tilde{S}_{\mathbf{j}}^{B,z}\right)$$

$$=\sin\psi\sum_{\mathbf{j}}\left(\tilde{S}_{\mathbf{j}}^{A,z}-\tilde{S}_{\mathbf{j}}^{B,z}\right)+\cos\psi\sum_{\mathbf{j}}\left(\tilde{S}_{\mathbf{j}}^{A,x}+\tilde{S}_{\mathbf{j}}^{B,x}\right)$$

$$=\sin\psi\sum_{\mathbf{j}}\left(2S-a_{\mathbf{j}}^{\dagger}a_{\mathbf{j}}-b_{\mathbf{j}}^{\dagger}b_{\mathbf{j}}\right)+\cos\psi\sum_{\mathbf{j}}\sqrt{\frac{S}{2}}\left(c_{\mathbf{j}}^{\dagger}+c_{\mathbf{j}}+b_{\mathbf{j}}^{\dagger}+b_{\mathbf{j}}\right).\quad\text{(S3)}$$

The second terms in the last line are linear in the magnon creation or annihilation operators, so that they are time-dependent with a factor of $e^{\pm i\,\hbar\omega_{1,2}(\mathbf{k})t+i\phi}$. Within the experimental measurement resolution, these contributions must be averaged to the zero. Besides, the initial phase ϕ in the time-dependent factor are equally distributed with $[0,2\pi]$, because the magnon are supposed to be thermally activated under the injector. After being averaged over the initial ϕ, the contributions must be zero too. Thus, we consider the contribution of the first term in equation (S3):

$$\sum_{\mathbf{j}}\left(S_{\mathbf{j}}^{A,x}+S_{\mathbf{j}}^{B,x}\right)=\sin\psi\sum_{\mathbf{j}}\left(\tilde{S}_{\mathbf{j}}^{A,z}-\tilde{S}_{\mathbf{j}}^{B,z}\right)=\sin\psi\sum_{\mathbf{j}}\left(2S-a_{\mathbf{j}}^{\dagger}a_{\mathbf{j}}-b_{\mathbf{j}}^{\dagger}b_{\mathbf{j}}\right)$$

A deviation of the spin density from its classical value is given by the magnon density:

$$\delta S^{x}\equiv\sum_{\mathbf{j}}\left(S_{\mathbf{j}}^{A,x}+S_{\mathbf{j}}^{B,x}\right)-2SN\sin\psi=-\sin\psi\sum_{\mathbf{j}}\left(a_{\mathbf{j}}^{\dagger}a_{\mathbf{j}}+b_{\mathbf{j}}^{\dagger}b_{\mathbf{j}}\right)$$

$$=-\sin\psi\sum_{\mathbf{k}}\left(\alpha_{\mathbf{k}}^{\dagger}\alpha_{\mathbf{k}}+\beta_{\mathbf{k}}^{\dagger}\beta_{\mathbf{k}}\right)$$

$$=-\sin\psi\sum_{\mathbf{k}}\left(\sinh^{2}\left(\frac{\xi_1}{2}\right)\gamma_{1,-\mathbf{k}}\gamma_{1,-\mathbf{k}}^{\dagger}+\cosh^{2}\left(\frac{\xi_1}{2}\right)\gamma_{1,\mathbf{k}}^{\dagger}\gamma_{1,\mathbf{k}}+\sinh^{2}\left(\frac{\xi_2}{2}\right)\gamma_{2,-\mathbf{k}}\gamma_{2,-\mathbf{k}}^{\dagger}\right.$$

$$\left.+\cosh^{2}\left(\frac{\xi_2}{2}\right)\gamma_{2,\mathbf{k}}^{\dagger}\gamma_{2,\mathbf{k}}\right)$$

$$=-\sin\psi\sum_{k}\left(\sinh^{2}\left(\frac{\xi_1}{2}\right)+\sinh^{2}\left(\frac{\xi_2}{2}\right)+\cosh\xi_1\,\gamma_{1,\mathbf{k}}^{\dagger}\gamma_{1,\mathbf{k}}+\cosh\xi_2\,\gamma_{2,\mathbf{k}}^{\dagger}\gamma_{2,\mathbf{k}}\right).\quad\text{(S4)}$$

In the third line of equation (S4), we omitted $\gamma^{\dagger}\gamma^{\dagger}$ and $\gamma\gamma$, which are time-dependent with a factor of $e^{\pm i2\,\hbar\omega_{1,2}(\mathbf{k})t+i\phi}$, for the same reason as in equation (S3). The omitted terms are time-

dependent with a factor of $e^{\pm i\,2E_{\dots}(\mathbf{k})t+i\phi}$, whose contributions must be averaged to zero in the experiments. The first two terms in the last line represent the spin-wave correction to the antiferromagnetic moment. We thus include them into *2SN*sinψ in the left hand side, and redefine the deviation of the spin density as:

$$\delta S^x \equiv \sum_{\mathbf{j}}\left(S_{\mathbf{j}}^{A,x}+S_{\mathbf{j}}^{B,x}\right) - 2SN\sin\psi + \sin\psi\sum_{\mathbf{k}}\left(\sinh^2\left(\frac{\xi_1}{2}\right)+\sinh^2\left(\frac{\xi_2}{2}\right)\right)$$
$$= -\sin\psi\sum_{\mathbf{k}}\cosh\xi_1\,\gamma^{\dagger}_{1,\mathbf{k}}\gamma_{1,\mathbf{k}} + \cosh\xi_2\,\gamma^{\dagger}_{2,\mathbf{k}}\gamma_{2,\mathbf{k}}$$

Note that the same signs in angular momenta of the two magnon modes; $\cosh\xi_1 > 0$, $\cosh\xi_2 > 0$. Since the two magnon modes have different group velocity $v_j(\mathbf{k}) = \nabla_{\mathbf{k}}\omega_j(\mathbf{k})$ ($j = 1,2$), the spin current density operator J_m is:

$$J_m = -\frac{\hbar}{V}\sin\psi\sum_{\mathbf{k}}\left(v_1(\mathbf{k})\cosh\xi_1\,\gamma^{\dagger}_{1,\mathbf{k}}\gamma_{1,\mathbf{k}} + v_2(\mathbf{k})\cosh\xi_2\,\gamma^{\dagger}_{2,\mathbf{k}}\gamma_{2,\mathbf{k}}\right)$$

We follow the same argument as Ref.[2], to obtain the spin Seebeck coefficient[2-5]:

$$\mathbf{J}_m = \mathbf{S}\cdot\nabla\mathrm{T}$$

$$\mathbf{S}(T) = \frac{\hbar^2\sin\psi}{k_BT^2}\sum_{j=1,2}\int_{BZ}\frac{dk_xdk_y}{(2\pi)^2}\mathbf{v}_j(\mathbf{k})\mathbf{v}_j(\mathbf{k})\cosh\xi_j\frac{e^{\hbar\omega_j(\mathbf{k})/k_BT}\omega_j(\mathbf{k})}{\eta_{j,k}\left(e^{\hbar\omega_j(\mathbf{k})/k_BT}-1\right)^2}$$

where $\eta_{j,k} = 1/\tau_{j,k}$ is the magnon relaxation time for the jth magnon branch and at magnon momentum k and the dispersion relation of the two magnon branches are:

$$\hbar\omega_1(\mathbf{k}) = \sqrt{(M_0+M_1)^2-(N_0-N_1)^2},$$
$$\hbar\omega_2(\mathbf{k}) = \sqrt{(M_0-M_1)^2-(N_0+N_1)^2},$$

Here,

$$M_0 = \frac{3JS}{2} + DS - \frac{DS}{2}\sin^2\psi, \quad M_1 = \frac{JS}{4}|f(\mathbf{k})|(-1+\cos 2\psi),$$

$$N_0 = \frac{DS}{2}\sin^2\psi, \quad N_1 = \frac{JS}{4}|f(\mathbf{k})|(1+\cos 2\psi),$$

$$f(\mathbf{k}) = \sum\nolimits_{m=1,2,3} e^{ik\hat{\mathbf{e}}_m}, \quad \mathbf{v}_j(\mathbf{k}) = \nabla_{\mathbf{k}}\omega_j(\mathbf{k}),$$

$$\cosh\xi_1 = \frac{M_0+M_1}{\hbar\omega_1(\mathbf{k})}, \quad \cosh\xi_2 = \frac{M_0-M_1}{\hbar\omega_2(\mathbf{k})}, \quad \sin\psi = \frac{h}{2(3J+D)S},$$

$$\hat{\mathbf{e}}_1 = (1,0), \quad \hat{\mathbf{e}}_2 = \left(-\frac{1}{2},\frac{\sqrt{3}}{2}\right), \quad \hat{\mathbf{e}}_3 = \left(-\frac{1}{2},-\frac{\sqrt{3}}{2}\right).$$

$\mathbf{v}_j(\mathbf{k})\mathbf{v}_j(\mathbf{k})$ is generally in the tensor-form and so is the Seebeck coefficient **S**. When considering only small k magnons around the Γ point where $\mathbf{v}_j(\mathbf{k}) \propto \hat{\mathbf{k}}$, $\mathbf{v}_j(\mathbf{k})\mathbf{v}_j(\mathbf{k})$ can be replaced by a scalar quantity:

$$\int \frac{dk_x dk_y}{(2\pi)^2}\mathbf{v}_j(\mathbf{k})\mathbf{v}_j(\mathbf{k}) \approx 1_{2\times 2}\int \frac{kdk}{6\pi} v_j^2(\mathbf{k})$$

Since

$$f(\mathbf{k}) = \sum_{m=1,2,3} e^{ik\hat{\mathbf{e}}_m}$$

We can expand $f(k)$ around the Γ point:

$$f(\mathbf{k}) = \sum_{m=1,2,3} e^{ik\hat{\mathbf{e}}_m} = \sum_{m=1,2,3}\left(1 + ik\hat{\mathbf{e}}_m + \frac{(ik\hat{\mathbf{e}}_m)^2}{2} + \mathrm{O}\left(ik\hat{\mathbf{e}}_m{}^3\right)\right)$$

$$= 3 + ik(\hat{\mathbf{e}}_1 + \hat{\mathbf{e}}_2 + \hat{\mathbf{e}}_3) - \frac{1}{2}\left(k_x{}^2 + \frac{1}{4}k_x{}^2 - \frac{\sqrt{3}}{4}k_x k_y + \frac{3}{4}k_y{}^2 + \frac{1}{4}k_x{}^2 + \frac{\sqrt{3}}{4}k_x k_y + \frac{3}{4}k_y{}^2\right) + \mathrm{O}\left(ik\hat{\mathbf{e}}_m{}^3\right)$$

Because $\hat{\mathbf{e}}_1 + \hat{\mathbf{e}}_2 + \hat{\mathbf{e}}_3 = 0$

So $f(k) = 3 - \frac{1}{2}\left(k_x{}^2 + \frac{1}{4}k_x{}^2 + \frac{3}{4}k_y{}^2 + \frac{1}{4}k_x{}^2 + \frac{3}{4}k_y{}^2\right) + \mathrm{O}\left(ik\hat{\mathbf{e}}_m{}^3\right) \approx 3 - \frac{3}{4}k^2$

With the above approximation, we are ready to simulate $V_{2\omega,0}$ as[2,6,7]:

$$V_{2\omega,0} = C * \left[\beta\left(\alpha I_{in}^2 + I_{gate}^2\right) * S\left(T = 2K + \beta\left(\alpha I_{in}^2 + I_{gate}^2\right)\right)\right]_{2\omega}$$

where, as stated in the main text, C is an overall constant, α is the relative strength between injector and gate electrode, β is the conversion efficiency from electrical current to temperature and $[\ldots]_{2\omega}$ means taking the second harmonic component.

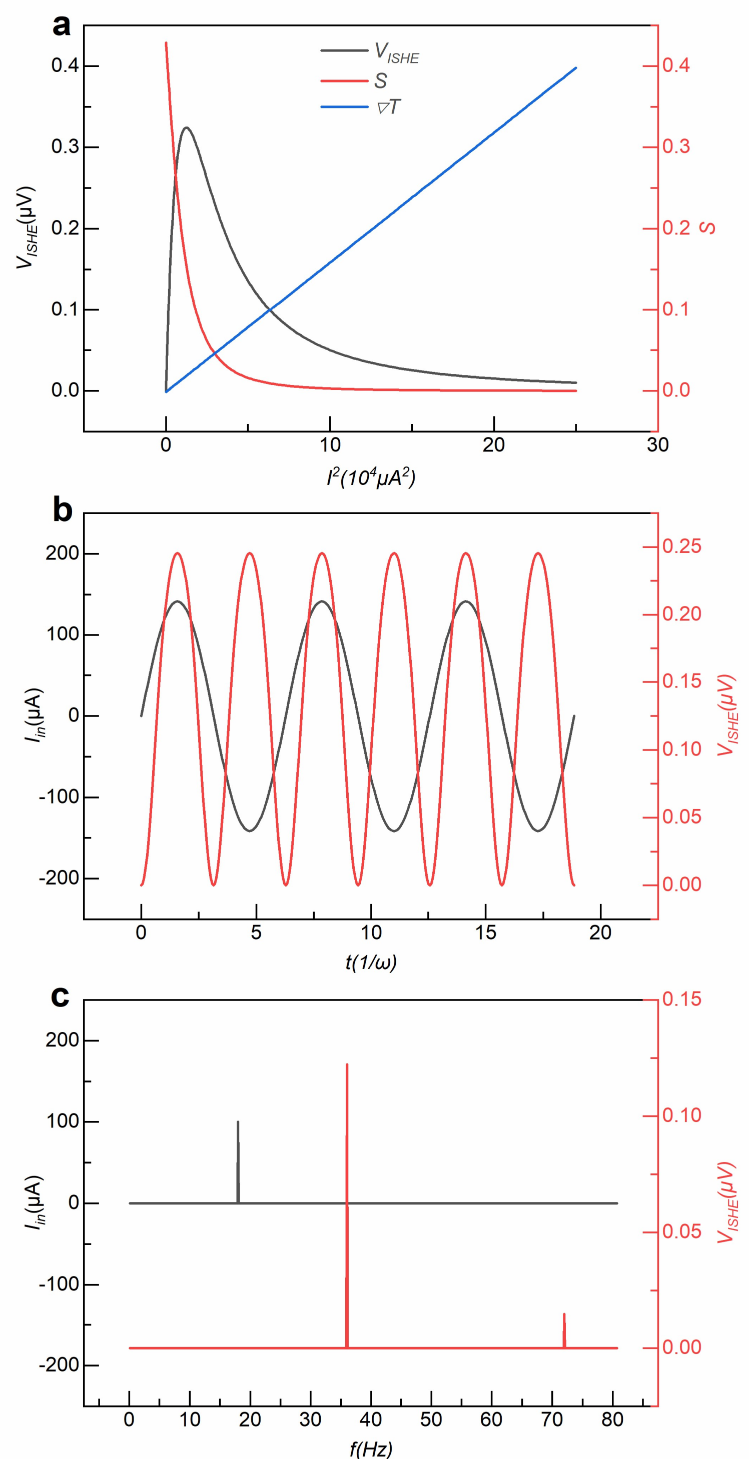

Fig. S4. Spin Seebeck effect in $MnPS_3$. (a) the simulated time-averaged inverse spin Hall voltage V_{ISHE} (black curve), the (1,1) component of the Seebeck coefficient tensor **S** (red curve), and the temperature gradience (blue curve) as a function of the square of the input current I^2

(proportional to the input power). (b) time dependent oscillations of AC injector current I_{in} (black curve) and the corresponding time dependence of V_{ISHE} (red curve). (c) frequency distribution of the AC injector current (black curve) and the V_{ISHE} (red curve). The values of the parameters C, α, β used in producing these figures follow those found in Fig. 4 in the main text.

One can also get a better understanding of the physical process by simulating the functional dependence of V_{ISHE}, $S(T)$ and ∇T on a general input current I^2 as can be found below in Fig. S4a. Under an AC injector excitation I_{in} of frequency 18.07Hz (as used in the experiment) and zero gate current, the temporal dependence and the frequency distribution of V_{ISHE} are shown in supplementary Fig. S4b and S4c, respectively. We can see that in this case, the second harmonic component $V_{2\omega,0}$ dominates the V_{ISHE} response, with a small fourth harmonic component.

S5. Quantitative analysis of the simulation

As discussed in the main text and in supplementary section S3 and S4, the simulation of $V_{2\omega,0}$ can be achieved via equation (4) with only three global parameters. Root means square deviation between the simulated curves and the experimental curves is calculated, and minimizing this deviation gives us the best values of the three global parameters: $C = 1.05 \times 10^{-26}\text{V}\cdot\text{s}/\hbar$, $\alpha = 0.25$ and $\beta = 1.69 \times 10^{-4}\text{K}/\mu\text{A}^2$ for device 2 shown in Figure 4 in the main text and $C = 1.14 \times 10^{-26}\text{V}\cdot\text{s}/\hbar$, $\alpha = 0.3$ and $\beta = 1 \times 10^{-4}\text{K}/\mu\text{A}^2$ for device 1 shown in Figure 3 (see the simulation and experimental data in Figure S5).

All the three parameters are complex functions of multiple factors including device dimensions, anisotropic heat conductance, specific heat of all the materials in the device, interfacial heat conductance, magnon-phonon interaction, external magnetic field, etc., just to name a few. It is beyond the main point of this article, e.g., the experimental realization of a van der Waals magnon valve, to exhaust all the possible factors that influence these three parameters, but we can still find that three parameters are highly reasonable and within the physical range with the following short discussion.

First of all, α is the ratio of the effective strength of the injector electrode over the gate electrode in terms of their influence to the detector-$MnPS_3$ interface. This ratio is device dependent, mainly depending on the device structure, the channel length and thickness, as well as the material of the channel and the electrodes. The overall effect of these factors can be experimentally measured in such an experiment: using the same detector, we can measure 1) the $V_{2\omega,0}$ caused by an AC current through the injector (with the gate electrode floating) and 2) the $V_{2\omega,0}$ caused by an AC current through the gate electrode (with the injector electrode floating). We called the former signal $V_{2\omega,in}$ and the later signal $V_{2\omega,gate}$. Since gate is closer to the detector than that of the injector, we can expect $\alpha < 1$, which indeed is the case for all our measurement using such device configurations. Quantitatively, we have carried out the before mentioned experiment at device 1 (also shown in Figure 3), at 9T and 2K, with an AC current 100μA, and we obtained $V_{2\omega,in}$ =19.28μV and $V_{2\omega,gate}$ = 29.92μV, so that $V_{2\omega,in}/V_{2\omega,gate}$ =0.6442. Note that for device 1, we have $C = 1.05 \times 10^{-26}\mathrm{V \cdot s}/\hbar$, $\alpha = 0.3$ and $\beta = 1 \times 10^{-4}\mathrm{K}/\mu\mathrm{A}^2$. We can put into the model the two sets of current parameters: 1) $I_{in} = 100\mu\mathrm{A}$ and $I_{gate} = 0\mu\mathrm{A}$ and 2) $I_{in} = 0\mu\mathrm{A}$ and $I_{gate} = 100\mu\mathrm{A}\ (AC)$. The simulated second harmonic detector signal is $V'_{2\omega,in} = 19.28\mu\mathrm{A}$ and $V'_{2\omega,gate} = 30.06\mu\mathrm{A}$, leading to $V'_{2\omega,in}/V'_{2\omega,gate} = 0.6414$, which agrees to the experimental value of 0.6442 very well. This shows the validity of the value of parameter α.

Now we turn to the discussion of parameter β. We can model β to be:

$$\beta = \frac{R * \tau}{C_{heat} * m}$$

where R is the resistance of the electrode (injector and gate), τ is a characteristic time for the system to achieve static conditions, C_{heat} is the specific heat of the $MnPS_3$ crystal, m is the mass of the crystal. Take the discussion on device 1 above, β = $1\times10^{-4}\mathrm{K}/\mu\mathrm{A}^2$, R is measured to be 1000Ω, the density and volume of $MnPS_3$ crystal is $\rho = 2.916 \times 10^3\mathrm{kg/m^3}$ and $V \sim 20\mathrm{nm}$ (thick) $\times$ $30\mu\mathrm{m}$(length) $\times$ $5\mu\mathrm{m}$(width) $= 3 \times 10^{-1}\ \mathrm{m}^3$, respectively, so the mass $m = \rho * V = 8.75 \times 10^{-9}\mathrm{mg}$, $C_{heat} = 0.5\ \mathrm{J \cdot mol^{-1}K^{-1}}$ [8]. If we consider the thermal mass of the Pt electrodes on the crystal, a rough estimation reveals that the $m_{pt} = 3.22 \times 10^{-9}\mathrm{mg}$, with $C_{heat} = 2.78\ \mathrm{J \cdot mol^{-1}K^{-1}}$ [9]. We conclude that τ would fall in the range of $4 \times 10^{-9}s$ to

$1.2 \times 10^{-8}s$, much smaller than the time constant of ~0.06s with respect to the measurement frequency 18.07Hz. This shows that the value of parameter β is within reasonable range.

The overall parameter C in equation (4) includes more factors in the physical process than the parameters α and β, for example mixing conductance between $MnPS_3$ and Pt. Note that the value of C includes the dimensionless prefactor of the magnon relaxation time $1/\eta_{j,k}$ (which is of order 10^{-8}) after summing contributions from all sublattice j and momentum k in the magnon Brillouin Zone, which brings in various effects including crystal quality, temperature and magnetic field. The fact that the C value from different $MnPS_3$ devices are close to each other shows that the device quality in our study is consistent.

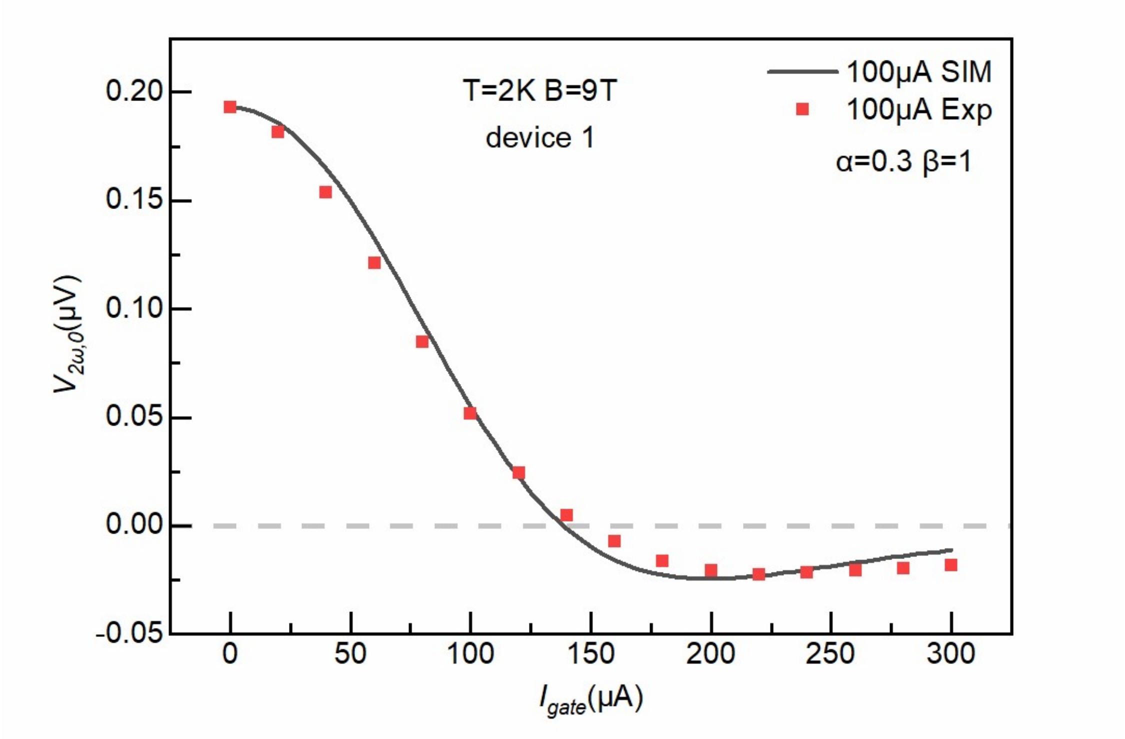

Fig. S5. Quantitative analysis of the simulation. $V_{2\omega,0}(I_{gate})$ of the $MnPS_3$ magnon valve device 1 at 2K and 9T, together with the simulation with the three parameters in equation (4) to be $C = 1.14 \times 10^{-26}\mathrm{V} \cdot \mathrm{s}/\hbar$, $\alpha = 0.3$ and $\beta = 1 \times 10^{-4}\mathrm{K}/\mu\mathrm{A}^2$.

Finally, we discuss the possibility to further reduce the parameters in the simulation one by one:

1) The overall parameter C in equation (4) mainly includes factors that relates the spin current injected into the detector Pt electrode to the voltage generated in the electrode; it also includes the dimensionless prefactor of the magnon relaxation time $1/\eta_{j,k}$ after summing contributions from all sublattice j and momentum k in the magnon Brillouin Zone, which brings in various effects including crystal quality, temperature and magnetic field. Thus this parameter is difficult to be removed yet very easily determined via fitting to an experimental curve.

2) The parameter β characterizes the effectiveness for the injection and gating current to be converted to temperature gradience in the device. In principle it could be obtained from parameters such as the resistivity of the Pt strips, the thermal conductivity of the Pt strips and the Au electrodes that are connected to the Pt strips (the 200nm Pt strips are connected to Cr/Au electrodes which extended into bonding pads for the devices), the thermal conductivity of the $MnPS_3$ crystal, as well as thermal resistivity of all the material interfaces. Considering the complexity of all these external parameters and our goal to derive a predictive effective model, the parameter β is best to be determined via fitting to an experimental curve.

3) The parameter α is the ratio of the effective strength of the injector electrode over the gate electrode in terms of their influence to the detector-$MnPS_3$ interface. This ratio is also device dependent, mainly depending on the device structure, the channel length and thickness, as well as the material of the channel and the electrodes. If equation (4) is a linear function of the temperature gradience, parameter α could be the easiest to be obtained from additional tests, such as the one we did above in this section (e.g., measure $V_{2\omega}$ for $I_{in} = 100\mu\text{A}$ and $I_{gate} = 0\mu\text{A}$ and then measure $V_{2\omega}$ again for $I_{in} = 0\mu\text{A}$ and $I_{gate,\text{ac}} = 100\mu\text{A}$). However, since the Seebeck coefficient S is an integral function that contains the temperature, and it is highly non-linear as shown in Supplementary Figure S4a, thus there is no simple relation between $V'_{2\omega,in}/V'_{2\omega,gate}$ and α. Accordingly, it is still the most straightforward way to use three parameters in the simulation.

S6. Comparison between experimental and simulated $V_{1\omega}$ and $V_{2\omega}$

For low AC current frequency, e.g. when the frequency ω of the injection current I_{in} is much lower than either the magnon frequency (~THz) and the response rate of the temperature of the device from the application of current (~GHz, see section S5), we can consider the magnon generation and detection process to be a non-equilibrium and static process. In such a process, the application of AC and DC current, the local change of sample temperature and the appearance of a detector signal can be considered instantaneous. Thus when I_{in} and I_{gate} is applied to the sample, the temperature T of $MnPS_3$ below the detector Pt can be approximated as:

$$T \approx T_{base} + \beta\left(\alpha I_{in}^2 e^{2i\omega t} + I_{gate}^2\right), \nabla T = \beta\left(\alpha I_{in}^2 e^{2i\omega t} + I_{gate}^2\right)$$

So we have:

$$J_S^x \propto |\mathbf{S}_S^x(\mathrm{T}) \cdot \hat{\mathbf{x}}| \nabla T = \left|\mathbf{S}_S^x\left(I_{in}, I_{gate}, t\right) \cdot \hat{\mathbf{x}}\right| \nabla T(I_{in}, I_{gate}, t)$$

Thus the first harmonic signal and second harmonic signal can be express as the following integral equations:

$$V_{1\omega,0} \propto J_{S,1\omega}^x \propto \int_{-\frac{\pi}{\omega}}^{\frac{\pi}{\omega}} e^{-i\omega t}\left|\mathbf{S}_S^x\left(I_{in}, I_{gate}, t\right) \cdot \hat{\mathbf{x}}\right| \nabla T(I_{in}, I_{gate}, t) dt$$

$$V_{2\omega,0} \propto J_{S,2\omega}^x \propto \int_{-\frac{\pi}{\omega}}^{\frac{\pi}{\omega}} e^{-i2\omega t}\left|\boldsymbol{S}_S^x\left(I_{in}, I_{gate}, t\right) \cdot \hat{\boldsymbol{x}}\right| \nabla T(I_{in}, I_{gate}, t) dt$$

From the above integral equations, we found finite $V_{2\omega}$ but vanishingly small $V_{1\omega}$, which agrees with the physics of thermal magnon excitation as well as our experimental measurements. See Figure S6 below for our experimentally measured first harmonic and second harmonic signal as a function of the angle of the in-plane magnetic field.

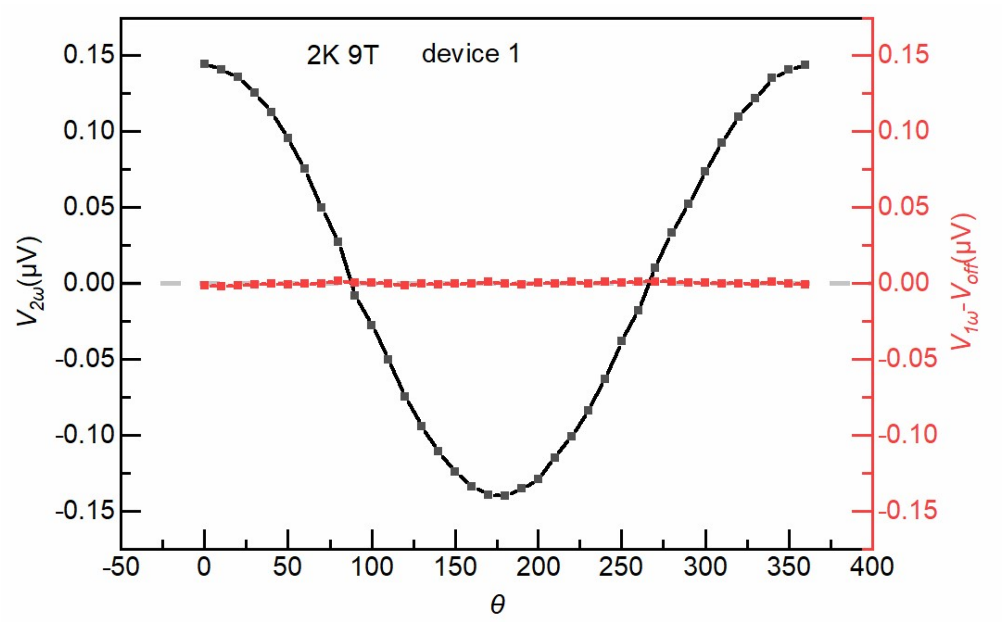

Fig. S6. Comparison between experimental and simulated $V_{1\omega}$ and $V_{2\omega}$. Magnetic field angular dependence of $V_{1\omega}$ and $V_{2\omega}$ for the $MnPS_3$ magnon valve devices. The magnitude of in-plane magnetic field is 9T and the temperature of the sample is at 2K. Here no gate current is applied and only an injection current of 100μA is applied to the injector. V_{off} is a constant offset voltage which accounts for the parasitic first harmonics signal that has no magnetic field dependence. The likely source of such signal includes parasitic inductance or capacitance between the measurement wires.

S7. Comparison between our work and recent work on $CrBr_3$

First of all, the channel materials are very different. $MnPS_3$ is a layered anti-ferromagnet with Ising-type anti-ferromagnetic coupling in the sample plane, while $CrBr_3$ is a 2D ferromagnet. This means that there is zero magnetic moment in each layer of $MnPS_3$, while each layer of $CrBr_3$ is magnetic. Such difference reflects strongly in the magnon spectra of the two materials [PRX 8, 011010 (2018), PRB 103, 024424 (2021)], and also reflects strongly in the existence of the magnetization at the Pt-$CrBr_3$ interface and the absence of which at the Pt-$MnPS_3$ interface, resulting in the observation of a large anomalous Nernst signal in $CrBr_3$ (PRB 101, 205407) and the absence of which in our work.

Second, the $R_{2\omega}$ vs. I_{in} curves are very different. We have plotted the $R_{2\omega}$ vs. I_{in} in Figure S13 for a couple of $MnPS_3$ devices below. In order to compare with the work on $CrBr_3$, the gate electrodes in our devices are floating during the measurement. It can be seen that the shape of the $R_{2\omega}$ vs. I_{in} of $MnPS_3$ device is very different from that of the $CrBr_3$ device shown in PRB 101, 205407. Interestingly, the shape of the $R_{2\omega}$ vs. I_{in} for $CrBr_3$ device has some resemblance with the $V_{2\omega}$ vs. I_{in} for our $MnPS_3$ devices, which would be a good topic for future works.

Third, the devices are very different. Our work realized the first diffusive magnon valves in which a gate current controls whether the injected signal can be detected or not, which readily enables digital logic operation; PRB 101, 205407 (2020) describe a non-local response curve for the input signal without any external gate control.

S8. The simulated crossing point I_0 vs. injection current I_{in}

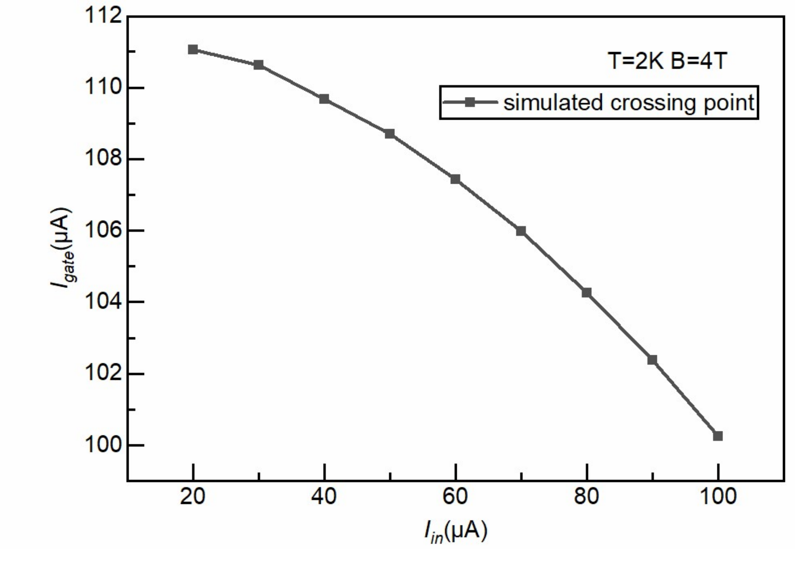

Fig. S8. The simulated crossing point I_0 (the first I_{gate} value for $V_{2\omega} = 0$) vs. injection current I_{in}. The simulation is based on parameters obtained from fitting to experimental data shown in Figure 4 in the main text.

S9. Nonlocal magnon signal detection with different $MnPS_3$ device geometries

Section S9 to S12 contain additional experimental evidences that excluded the possibility of local spin Seebeck effect as well as anomalous Nernst effect in the $MnPS_3$ magnon valves.

First of all, to clarify whether the heat is carried by phonon or by magnon, we have fabricated a non-local device with a number of electrodes on $MnPS_3$. As depicted in Fig. S9 below, we name four of the electrodes as Detector 1, Injector, oxidized Cu strip and Detector 2, respectively. All electrodes are made from Pt except for the oxidized Cu strip. The oxidized Cu strip is made from 10nm thick copper without any protection capping layer and then is exposed to ambient condition for oxidation. We have made sure that the Cu strip was conductive right after the deposition and not conductive after oxidation. The intention of the oxidation is to reduce the thermal conductivity of the copper strip to 4 W/m*K[10], so that it is much lower than a Pt electrode in terms of thermal conductivity (72W/m*K for Pt). The oxidized copper strip merely acts as surface absorbates which only affect the top surface of $MnPS_3$ and would not act as a strong heat sink. In another word, the oxidized Cu strip should perturb magnon transport much more than phonon transport in $MnPS_3$, since the in-plane to out-of-plane ratio of magnetic coupling strength is 405:1 [1] while the in-plane to out-of-plane ratio of thermal conductivity is only 6:1 [11]. The strong in-plane versus out-of-plane anisotropy in the magnetic exchange suggests that the Pt detectable magnon transport goes through only a few top layers of the sample, while the phonon transport generally goes through the whole layers of the sample. Being only the surface absorbates, the oxidized copper strip is expected to perturb the magnon transport dramatically, while the phonon transport remains robust against such perturbation.

An AC signal is applied through the Injector electrode, and the signal is measured simultaneously from Detector 1 and Detector 2. We found strong signal from Detector 1 (right next to the Injector) and no signal from Detector 2 (the oxidized copper strip is between Detector 2 and the Injector). Since the temperature gradient between Injector and Detector 2 should be finite as the case for Detector 1, which is confirmed by finite element analysis (see Fig. S9c). The absence of the non-local inverse spin Hall signal from Detector 2 proves that phonon transport is not the cause of the non-local signal from the Detector electrode.

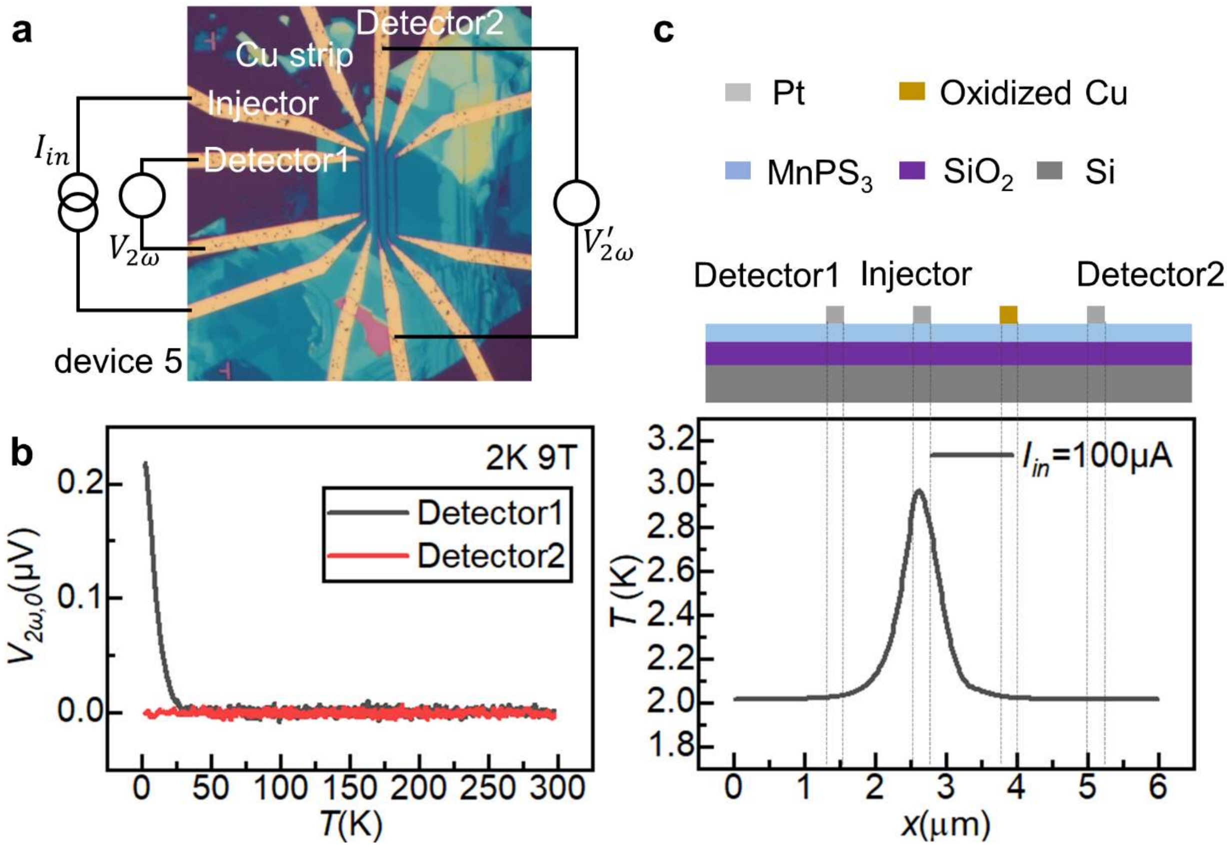

Fig. S9. Nonlocal magnon signal detection with different $MnPS_3$ device geometries. (a) Schematic of nonlocal measurement on a $MnPS_3$ device with oxidized Cu strip. (b) Temperature dependence of $V_{2\omega}$ at $\theta = 0$ for the detector located at the left or right side of the oxidized Cu strip. (c) Finite element analysis of the temperature distribution in $MnPS_3$ device with oxidized Cu strip.

S10. Absence of the anomalous Nernst effect in $MnPS_3$ magnon valve

To quantify the effect of the anomalous Nernst effect in our experimental system, we have measured the non-local second harmonic signal with an applied magnetic field of up to 14 T rotated in the *x*-*z* plane (see inset in Fig. S10 below). Since there is finite temperature gradient along the *x* axis from our device configuration as shown from the finite element analysis, the temperature gradient along *x* also induces the Hall voltage along the *y* axis in the presence of the magnetization along the *z* axis (ANEx). It's considered that ANEx and ANEz are of similar

magnitude[12], where ANEz refers to the Hall voltage along y induced by the temperature gradient along z in the presence of the magnetization along the x axis.

The angle of the magnetic field with respect to the z axis is marked as φ. An injection current of 100 μA is applied to the injector of our $MnPS_3$ device. We can see from Fig. S10a that the data fits well to a sinφ function, in which the signal is zero when the magnetic field is along the z axis (perpendicular to the sample plane). From Fig. S10a one can also see that only the magnetic field component along the x axis could produce non-zero non-local second harmonic signal. Figure S10b shows minimal magnetic-field dependence of the non-local signal with the magnetic field along the z axis (i.e., φ=0). This data proves unambiguously the absence of anomalous Nernst effect with magnetic field perpendicular to the $MnPS_3$/Pt interface (ANEx), because the finite element calculation shows a finite temperature gradient along x. The absence of ANEx points to the absence of ANEz[12].

In fact, $MnPS_3$ is a layer antiferromagnet where the spin within one Mn atomic layer is aligned antiferromagnetically with a coupling constant J that amount to about 106T of magnetic field, which far exceeds the magnetic field applied in the experiment. It is natural that the $MnPS_3$/Pt interface remains non-magnetized. We have added this data in Supplementary Figure S10, with a short description.

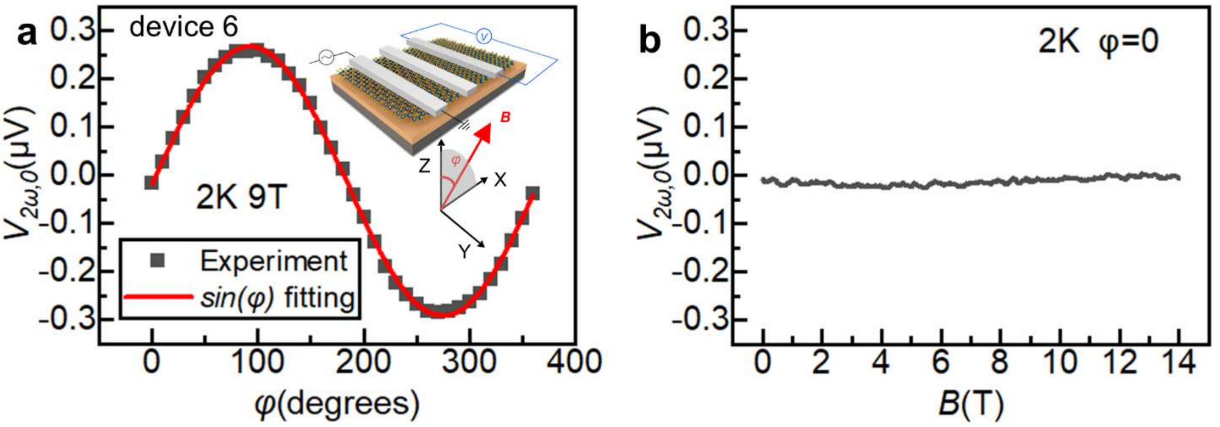

Fig. S10. The absence of the anomalous Nernst effect in $MnPS_3$ magnon valve. (a) The non-local second harmonic signal as a function of angle φ between the external magnetic field (B=9T) and the z direction, angle φ is determined the same as shown in the inset.(b) The absence of magnetic field dependence of $V_{2\omega}$ at $\varphi = 0$.

S11. The heater-detector distance-dependent signal and temperature in $MnPS_3$ device.

We have measured the distance dependence of $V_{2\omega,0}$ (black dots in Fig. S11) and the experimental data is fitted to $V_{2\omega} = \frac{C_0}{\lambda} * \frac{\exp(d/\lambda)}{1-\exp(2d/\lambda)}$ [13], where C_0 is a factor characterizing the magnitude of the second harmonic signal, λ is the decay length of the diffusive magnons. The fitting gives $C_0 = -2.4 \pm 0.2 \mu V \cdot \mu m$ and λ = 3300nm±200nm, which is consistent with previous report in the literature (e.g. λ~2800 nm for 16-nm $MnPS_3$, 1100 nm for 8-nm $MnPS_3$ [14]). As shown in Fig. S11, this decay length (red curve) is much longer than a decay length of the temperature gradient from the finite element calculation (blue curve) that represents how far the phonon carries the heat in space. Thus, the longer decay length in the heater-detector distance dependence suggests that the signal cannot be explained by the phonon transport.

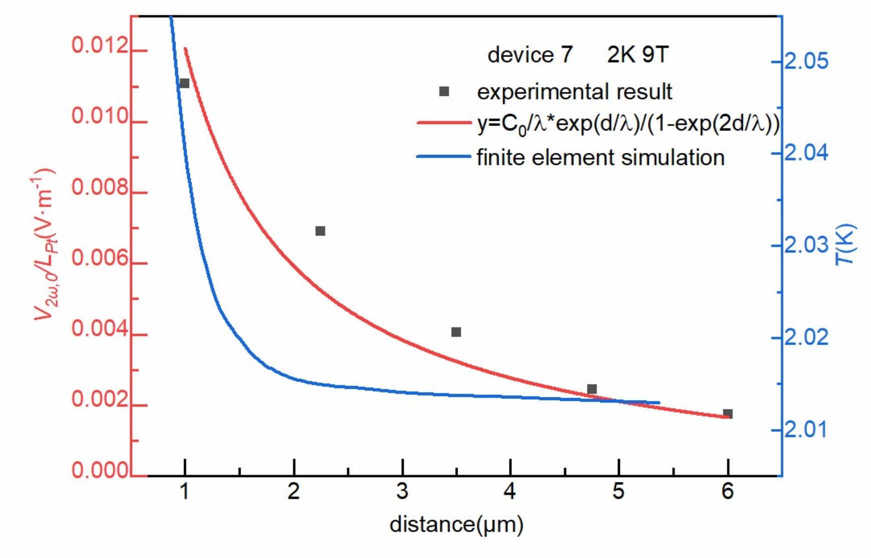

Fig. S11. The heater-detector distance-dependent signal and temperature in $MnPS_3$ device. Left axis: The distance-dependent non-local second harmonic signal $V_{2\omega,0}$ and relaxation length fitting[13] at 2K and 9T; Right axis: The finite element simulation of distance-dependent temperature of $MnPS_3$ under the same device configuration. The decay length of the second harmonic thermal magnon signal is measured to be 3300nm±200nm.

S12. Operation of a $MnPS_3$ magnon valve with different device geometries

Two different device geometries, namely, the injector-gate- detector configuration as well as the injector-detector-gate configuration is tested. We found that in the injector-detector-gate configuration, the general behavior is similar (see Fig. S12b). Finite element analysis shows that the variation of the temperature gradient is also similar for the gate located at the right or left side of the detector electrode (see Fig. S12c&d), while the temperature of the $MnPS_3$ below the detector electrode is slightly higher for the case of the injector-detector-gate configuration, which may be the cause of the slight difference in $V_{2\omega}$ vs. I_{gate} observed experimentally.

The parameters used in the finite element analysis are listed below:

Pt	Conductivity	8.9E6[S/m]	COMSOL Material database
	thermal conductivity	71.6[W/(m*K)]	COMSOL Material database
$MnPS_3$	in-plane thermal conductivity	6.3[W/(m*K)]	ACS Nano,14, 2424−2435(2020)
	through-plane thermal conductivity	1.1[W/(m*K)]	ACS Nano,14, 2424−2435(2020)
SiO_2	thermal conductivity	1.38[W/(m*K)]	CRC Handbook of Chemistry and Physics (92nd ed.).p12.213
Si	thermal conductivity	130[W/(m*K)]	COMSOL Material database
$MnPS_3/SiO_2$	through-plane thermal resistance	5E-7[K*m^2/W][#]	Computational Materials Science, 142, 1−6 (2018)
Pt/$MnPS_3$	through-plane thermal resistance	1.4E-7[K*m^2/W][§]	PHYSICAL REVIEW B 101, 205407 (2020)

Table S1. The parameters used in the finite element analysis. [#]There is no data found for $MnPS_3/SiO_2$ in the literature, we used value from through-plane thermal resistance between MoS_2/SiO_2 instead. [§]There is no data found for Pt/$MnPS_3$, we used estimated value for $CrBr_3$/Pt in the literature.

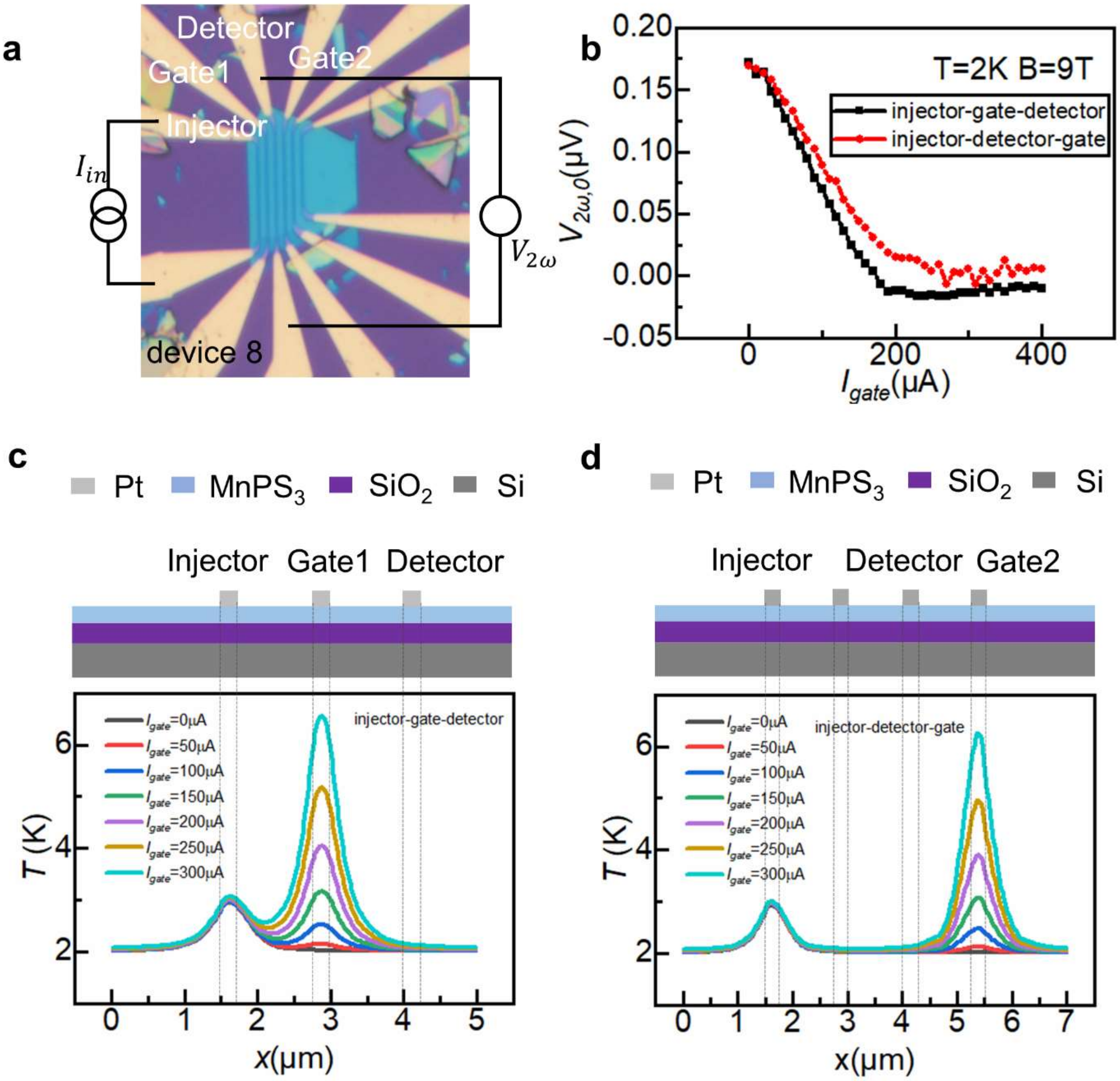

Fig. S12. Operation of a $MnPS_3$ magnon valve with different device geometries. (a) Schematics of nonlocal measurement on a $MnPS_3$ device with different gates. (b) $V_{2\omega,0}$ versus DC gate current I_{gate} at B = 9T and temperature of 2K with different geometries. (c)(d) Finite element analysis of the temperature distribution in $MnPS_3$ device for the gate located at the left (c) or right (d) side of the detector electrode.

S13. $R_{2\omega}$ vs. I_{in} for different $MnPS_3$ devices with zero gate current

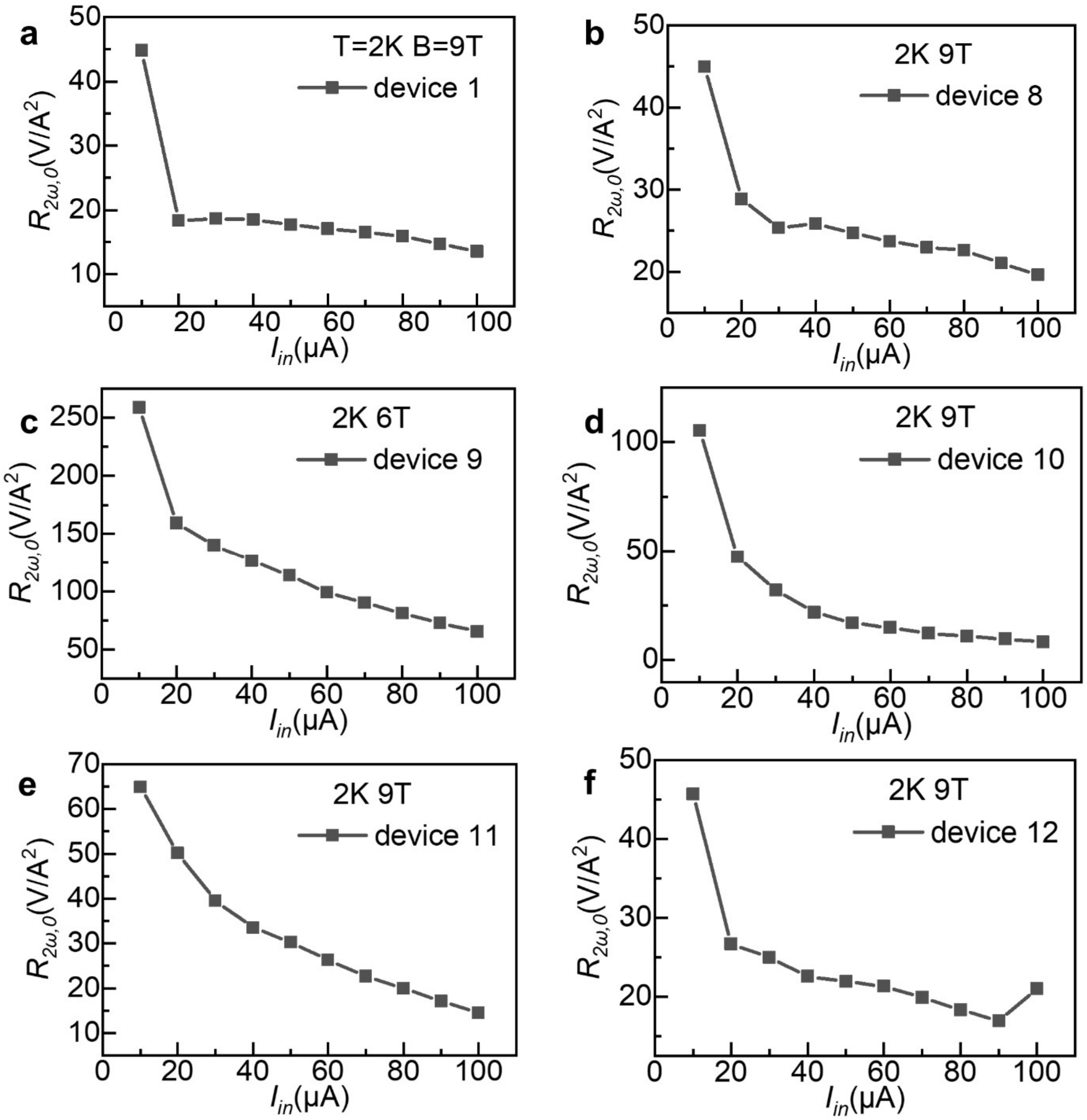

Fig. S13. The $R_{2\omega}$ vs. I_{in} for different $MnPS_3$ devices with zero gate current.

S14. $V_{2\omega}$ vs. I_{in} for different $MnPS_3$ devices with zero gate current

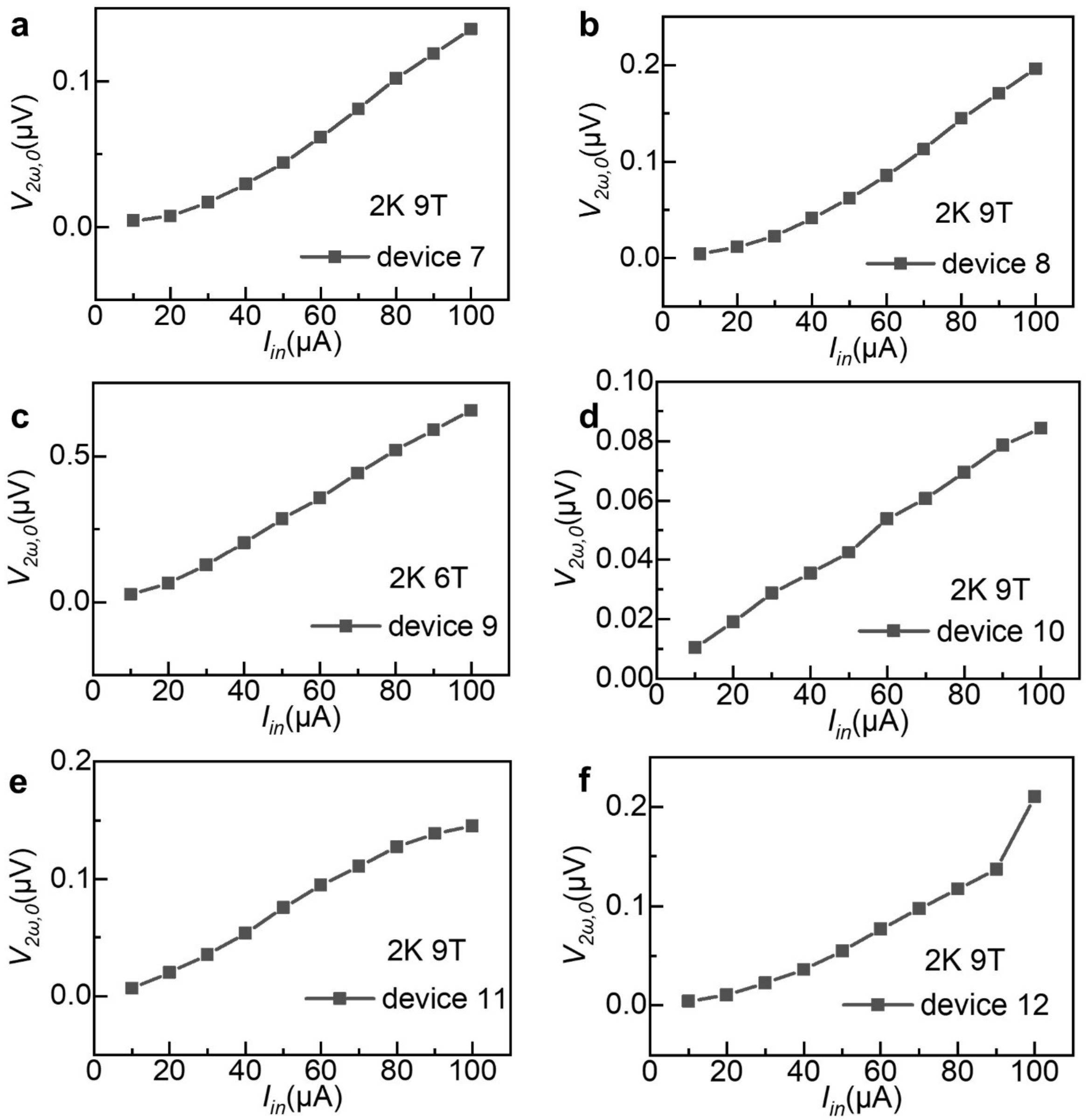

Fig. S14. The $V_{2\omega}$ vs. I_{in} curves for the same set of $MnPS_3$ devices as shown in Fig. S13 with zero gate current.

S15. Stability test of few-layer $MnPS_3$ crystals and devices

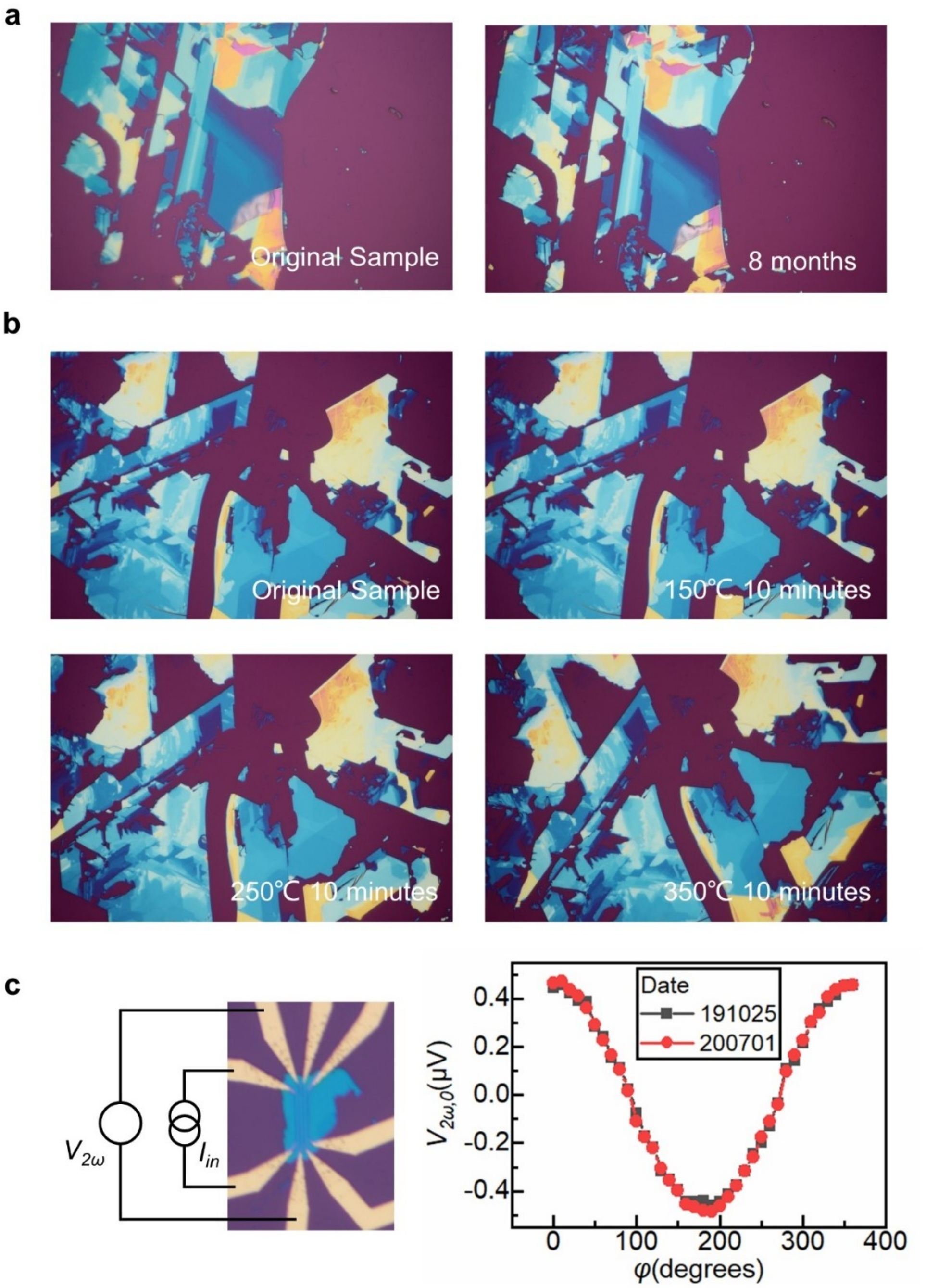

Fig. S15. Stability test of few-layer $MnPS_3$ crystals and devices. (a) the optical micrograph of few-layer $MnPS_3$ on SiO_2 substrate right after exfoliation and 8 months after exfoliation. (b) Optical micrographs of few-layer $MnPS_3$ on SiO_2 substrate before heating and after heating to 150, 250 and 350°C for 10 minutes in air. (c) The device performance of our $MnPS_3$ magnon device right after fabrication and after 8 months.

Reference

1. Wildes, A. R., Roessli, B., Lebech, B. & Godfrey, K. W. Spin waves and the critical behaviour of the magnetization in $MnPS_3$. *J. Phys.: Condens. Matter* **10**, 6417-6428 (1998).
2. Rezende, S. M., Rodriguez-Suarez, R. L. & Azevedo, A. Theory of the spin Seebeck effect in antiferromagnets. *Phys. Rev. B* **93**, 014425 (2016).
3. Poonja, S., Patel, S., Henry, L. & Roorda, A. Dynamic visual stimulus presentation in an adaptive optics scanning laser ophthalmoscope. *Journal Of Refractive Surgery* **21**, S575-S580 (2005).
4. Cornelissen, L. J. *et al.* Magnon spin transport driven by the magnon chemical potential in a magnetic insulator. *Phys. Rev. B* **94**, 014412 (2016).
5. Duine, R. A., Brataas, A., Bender, S. A. & Tserkovnyak, Y. in *Universal Themes of Bose-Einstein Condensation* **Ch. 26**, 505-524 (2017).
6. Sanders, D. J. & Walton, D. Effect of magnon-phonon thermal relaxation on heat transport by magnons. *Phys. Rev. B* **15**, 1489-1494 (1977).
7. Agrawal, M. *et al.* Direct measurement of magnon temperature: new insight into magnon-phonon coupling in magnetic insulators. *Phys. Rev. Lett.* **111**, 107204 (2013).
8. Takano, Y. *et al.* Magnetic properties and specific heat of MPS_3 (M=Mn, Fe, Zn). *J. Magn. Magn. Mater.* **272**, E593-E595 (2004).
9. Boerstoel, B. M., Zwart, J. J. & Hansen, J. Specific Heat of Palladium, Platinum,Gold and Copper Below 30k. *Physica* **54**, 442-458 (1971).
10. Kusiak, A. *et al.* CuO thin films thermal conductivity and interfacial thermal resistance estimation. *European Physical Journal-Applied Physics* **35**, 17-27 (2006).
11. Kargar, F. *et al.* Phonon and Thermal Properties of Quasi-Two-Dimensional $FePS_3$ and $MnPS_3$ Antiferromagnetic Semiconductors. *ACS Nano* **14**, 2424-2435 (2020).
12. Liu, T. *et al.* Spin caloritronics in a CrBr3-based magnetic van der Waals heterostructure. *Phys. Rev. B* **101** (2020).
13. Cornelissen, L. J. *et al.* Long-distance transport of magnon spin information in a magnetic insulator at room temperature. *Nat. Phys.* **11**, 1022-1026 (2015).
14. Xing, W. Y. *et al.* Magnon Transport in Quasi-Two-Dimensional van der Waals Antiferromagnets. *Phys. Rev. X* **9**, 011026 (2019).
